# Immune checkpoint inhibition combined with targeted therapy using a novel virus-like drug conjugate induces complete responses in a murine model of local and distant tumors

**DOI:** 10.1007/s00262-023-03425-3

**Published:** 2023-03-30

**Authors:** Ruben V. Huis in ‘t Veld, Sen Ma, Rhonda C. Kines, Anneli Savinainen, Cadmus Rich, Ferry Ossendorp, Martine J. Jager

**Affiliations:** 1 Department of Ophthalmology, Leiden University Medical Centre (LUMC), Leiden, The Netherlands; 2 Department of Radiology, Leiden University Medical Centre (LUMC), Leiden, The Netherlands; 3 Department of Immunology, Leiden University Medical Centre (LUMC), Leiden, The Netherlands; 4 Aura Biosciences, Cambridge, MA USA

**Keywords:** Cancer, Metastasis, AU-011 treatment, Immunotherapy, Immune checkpoint inhibition, Immunogenic cell death

## Abstract

Metastases remain the leading cause of cancer-related death worldwide. Therefore, improving the treatment efficacy against such tumors is essential to enhance patient survival. AU-011 (belzupacap sarotalocan) is a new virus-like drug conjugate which is currently in clinical development for the treatment of small choroidal melanoma and high-risk indeterminate lesions in the eye. Upon light activation, AU-011 induces rapid necrotic cell death which is pro-inflammatory and pro-immunogenic, resulting in an anti-tumor immune response. As AU-011 is known to induce systemic anti-tumor immune responses, we investigated whether this combination therapy would also be effective against distant, untreated tumors, as a model for treating local and distant tumors by abscopal immune effects. We compared the efficacy of combining AU-011 with several different checkpoint blockade antibodies to identify optimal treatment regimens in an in vivo tumor model. We show that AU-011 induces immunogenic cell death through the release and exposure of damage-associated molecular patterns (DAMPs), resulting in the maturation of dendritic cells in vitro. Furthermore, we show that AU-011 accumulates in MC38 tumors over time and that ICI enhances the efficacy of AU-011 against established tumors in mice, resulting in complete responses for specific combinations in all treated animals bearing a single MC38 tumor. Finally, we show that AU-011 and anti-PD-L1/anti-LAG-3 antibody treatment was an optimal combination in an abscopal model, inducing complete responses in approximately 75% of animals. Our data show the feasibility of combining AU-011 with PD-L1 and LAG-3 antibodies for the treatment of primary and distant tumors.

**Supplementary Information:**

The online version contains supplementary material available at 10.1007/s00262-023-03425-3.

## Introduction

Cancer is one of the major health problems worldwide. While the treatment of cancer with photodynamic therapy (PDT) has been shown to have anti-tumor activity in some types of local tumors, it has not had a major impact in the treatment of metastatic disease. The tumor specificity of PDT is determined by the type and location of the photosensitizer (PS) at the time of light activation and the area illuminated by the light source. In cases of cutaneous cancer lesions, a PS is often administered topically; in other cases, like the treatment of intra-ocular lesions, it may be injected intravenously, after which the PS passively and nonspecifically localizes in the tumor. However, many PS show little selectivity for malignant cells and often induce toxicities in various non-malignant cells. Several carriers for PS that may increase cancer cell specificity have been investigated, including monoclonal antibodies, liposomes, polymeric nanoparticles and other types of nanoparticle formulations [1–3]. Although these carriers generally enhance PS distribution to the tumor area, they often require functionalization with targeting moieties to increase cancer cell selectivity within the tumor. Such targeting may be achieved with PS-conjugate antibodies, that target markers enriched in the tumor and its vasculature with demonstrated efficacy in preclinical models [4–7]. Recently, it was shown that virus-like particles (VLPs) derived from human papillomavirus (HPV) preferentially bind to cancer cells in vitro and in vivo [8]. The selectivity for cancer cells is based on binding of the VLPs to modified heparan sulfate proteoglycans (HSPGs), which have been shown to be a primary factor required for HPV entry into cells [9–11]. As the tumor microenvironment is enriched in specifically modified HSPGs [12–15], VLPs may be suitable carriers for tumor-targeting agents in cancer therapy. Virus-like drug conjugates (VDCs) are a novel class of targeted therapy that is based on this selectivity of HPV-derived VLPs for cancer cells. AU-011 is a first in class light-activated VDC which consists of an HPV-derived VLP with approximately 200 molecules of a novel photosensitizer (IRDye 700DX), and can be activated using a standard PDT laser. Administration of AU-011 followed by PDT laser activation has shown anti-cancer activity in several human tumor cell lines in vitro, and has also shown to induce cytotoxicity in 92.1 Uveal Melanoma (UM) cells inoculated subcutaneously in nude mice as well as tumor necrosis in intra-ocular orthotopic UM xenografts in rabbits [16]. This treatment is being evaluated in two clinical trials for the treatment of choroidal melanoma and indeterminate choroidal pigmented lesions. A Phase 1b/2 trial was recently completed utilizing intravitreal administration (NCT03052127) and a Phase 2 trial utilizing suprachoroidal administration is ongoing (NCT04417530). In addition, AU-011 is being developed to treat other malignant tumors such as non-muscle invasive bladder cancer, and choroidal metastasis of other tumors (e.g., lung, breast, colon).

In patients, metastatic tumors remain the primary cause of cancer-related mortality and morbidity [17, 18]. Therefore, increasing efficacy against such tumors is vital to improve patient outcomes and survival. In recent years, novel insights in cancer immunotherapy have produced several types of immune checkpoint inhibitors (ICIs) that are relatively successful against metastatic tumors [19]. However, not all patients respond to this type of treatment and often develop tumor resistance, in spite of efforts to enhance the efficacy by addressing additional immunotherapeutic targets. The efficacy of ICIs may be aided by a cancer vaccine-mediated induction [20], or adoptive transfer [21], of tumor-specific T cells [22]. Furthermore, PDT was shown to synergize with ICIs, inducing an abscopal effect that enhanced survival of treated animals [23–26]. Together with the notion that ICIs may be aided by the induction of tumor-specific T cells [22], these results suggest that PDT in cancer treatment could function as an in situ vaccination strategy that may be further enhanced by ICIs. It has been previously described that AU-011 combined with checkpoint blockade antibodies led to complete or partial tumor responses, respectively, against subcutaneous TC-1 tumors in mice, protecting animals from further tumor challenge [27].

In the present study, we compared the efficacy of AU-011 combined with several ICIs to treat not only primary local but also secondary distant tumors in mice. To determine the optimal combination regimen, we tested the clinically used antibodies against CTLA-4 and PD-L1, as well as antibodies against LAG-3. Our recent high-dimensional mass cytometry analyis [28] showed that PD-L1 blockade in MC38 tumor-bearing mice selectively induces expansion of tumor-infiltrating T cells that co-express activating (ICOS) and inhibitory (LAG-3, PD-1) molecules. We and others have previously shown that therapeutic co-targeting of PD-L1 and LAG-3/ICOS enhances the tumor growth inhibition of either antibody used in monotherapy [28]. For this reason, we evaluated the potential of AU-011 combined with anti-PD-L1 and anti-LAG-3 antibodies using a metastatic murine tumor model of colon cancer. We investigated the ability of AU-011 to induce cytotoxicity in a panel of cancer cell lines in vitro and examined whether the treatment displays preference for cancer over APCs. We further explored the capacity of AU-011 to induce immunogenic cell death in cancer cells by measuring the release and exposure of damage-associated molecular patterns (DAMPs), and their ability to facilitate subsequent dendritic cell maturation. In vivo, we explored the tumor distribution of AU-011, the antitumor efficacy of the combination of AU-011 with several ICI, and the feasibility of AU-011 combined with ICI treatment in a model of metastatic tumors.

## Materials and methods

### Cells

The Murine Colon 38 (MC38) carcinoma cell line and the murine colon carcinoma cell line CT26 were provided by Mario Colombo and the murine TC-1 cell line, a lung fibroblast transduced with retrovirus to express HPV16 E6 and E7 oncoproteins in addition to the activated human c-Ha-ras oncogene, was a gift from T.C. Wu (John Hopkins University, Baltimore, MD). For flow cytometry purposes, MC38 cells were transduced with lentivirus to express Cyan Fluorescent Protein (CFP) and subsequently sorted on a BD FACSARIA II based on CFP^+^ to obtain MC38CFP. With the exception of the colon cancer panel comparison, cells were cultured in Iscove's Modified Dulbecco's Medium (IMDM; Lonza, Basel, Switzerland) supplemented with 8% Fetal Calf Serum (Greiner, Austria), 2 mM glutamine (Gibco, Landsmeer, The Netherlands), 100 IU/mL penicillin/streptomycin (Gibco, Landsmeer, The Netherlands) and 25 μM 2-mercaptoethanol (Sigma-Aldrich, Zwijndrecht, The Netherlands). For the colon cancer panel comparison (Fig. 2c-d), the cells were cultured in DMEM (Invitrogen, Waltham, MA, USA) supplemented with 10% fetal bovine serum (FBS). The human colon cancer lines are part of the NCI-60 cell line panel and were acquired from the Developmental Therapeutics Program at the National Cancer Institute (Frederick, MD, USA). MC38 cells in this panel comparison were obtained from Dr. James Hodges (NCI, Bethesda, MD, USA [29]) and cultured in DMEM supplemented with 10% FBS, 1 mmol/L sodium pyruvate, 2 mmol/L L-glutamine, 0.1 mmol/L nonessential amino acids, 10 mmol/L HEPES, and 50 µg/mL gentamycin (all components were obtained from Invitrogen, Waltham, MA, USA). CT26 cells in this panel comparison were acquired from TD2 (Scottsdale, AZ, USA) and cultured in RPMI (Invitrogen, Waltham, MA, USA) supplemented with 10% FBS. D1 Dendritic cells (D1DCs) [30] were cultured as described [31] in D1DC culture medium consisting of Iscove's Modified Dulbecco's Medium (Lonza, Basel, Switzerland) supplemented with 8% Fetal Calf Serum (Greiner, Austria), 100 IU/mL penicillin (Gibco, Landsmeer, The Netherlands), 2 mM glutamine (Gibco, Landsmeer, The Netherlands) and 25 μM β-mercaptoethanol (Sigma-Aldrich, Zwijndrecht, The Netherlands). All cells used were regularly mouse antibody production (MAP)-tested as well as tested for mycoplasma before the start of experiments and maintained at 37 °C and 5% CO_2_ in an incubator (Panasonic,’s- Hertogenbosch, The Netherlands), unless indicated otherwise.

### AU-011 internalization, binding and preferential association

To determine the internalization and binding of AU-011 in mono-culture, 5 × 10^4^ MC38 cells, 4 × 10^4^ CT26 cells or 3.5 × 10^4^ TC-1 cells were seeded in 24-well plates (Corning, Glendale, CA, USA) in culture medium and incubated overnight at 37 °C in 5% CO_2_. Cells were then incubated in culture medium containing 3–3000 pM of AU-011 for a specified time at 4 °C for binding only and at 37 °C for internalization in addition to binding. After incubation, the cells were washed three times with phosphate-buffered saline (PBS) and fixed in PBS with 1% formalin (J.T. Baker, Landsmeer, The Netherlands) at 4 °C for 15 min. Following this, the fixative was washed three times with PBS, after which the cells were reconstituted in Fluorescence-Activated Cell Sorting (FACS) buffer (PBS with 0.5% Bovine Serum Albumin (BSA) and 0.02% sodium azide). After this, the binding and internalization were determined by measuring the fluorescence of the photosensitizer using flow cytometry on a Cytek Aurora 3-Laser flow cytometer (Cytek, Fremont, CA, USA). The binding and internalization of AU-011 in co-culture was determined by seeding 4 × 10^4^ MC38CFP in 24-well plates and overnight incubation at 37 °C and 5% CO_2_. The next morning, the cancer cells were counted and an equal number of D1DCs was added to the well. The resulting co-culture was subsequently incubated with 300 pM AU-011 for 4 h at 37 °C, washed three times in PBS and reconstituted in FACS buffer. To differentiate MC38CFP from D1DCs, D1DCs were stained with anti-CD11c-PE (Clone HL3; BD Biosciences, New Jersey, USA), followed by analysis on a Cytek Aurora 3-Laser flow cytometer.

### Subcellular localization of AU-011 with fluorescence microscopy

To determine the subcellular location of AU-011, MC38 cells were seeded in 8-chamber polystyrene slides with removable well (Thermofisher, Landsmeer, The Netherlands) at 10^4^ cells per chamber and incubated overnight at 37 °C and 5% CO_2_. The cells were then incubated with 300 pM AU-011 for 4 h at 37 °C, washed three times with PBS and stained with CD44-FITC (Clone IM7; Thermofisher, Landsmeer, The Netherlands) in FACS buffer at 4 °C for 30 min. Samples were then washed three times in PBS and fixed in PBS with 1% formalin (J.T. Baker, Landsmeer, The Netherlands) at 4 °C for 15 min. The fixative was then washed three times in PBS, after which the wells were removed from the slide according to the manufacturers’ instructions. Cover slips were subsequently mounted on the slides with Vectashield mounting medium with DAPI (Vector Labs, Oxfordshire, UK) and samples were analyzed on a Leica Coolsnap DMRA fluorescence microscope.

### *AU-011 *in vitro* cytotoxicity in mono- and co-culture*

For AU-011 activated with a PDT laser in mono-culture, 5 × 10^4^ MC38 cells, 4 × 10^4^ CT26 cells or 3.5 × 10^4^ TC-1 cells were seeded in 24-well plates (Corning, USA) in culture medium and incubated overnight at 37 °C in 5% CO_2_. Unless indicated otherwise, cells were then incubated in culture medium containing 300 pM AU-011 for 4 h, washed in PBS, provided with fresh culture medium and subsequently illuminated with 690 nm light emitted from a custom liquid fiber-coupled red diode laser system (LED diode laser, CNI Laser, Changchun, China) at 400 mW/cm^2^ for 25 J/cm^2^. Cells were then kept in an incubator for 18 h, collected in FACS buffer, stained with 0.5 µM of the viability marker 4′,6-diamidino-2-phenylindole (DAPI) (Sigma-Aldrich, Zwijndrecht, The Netherlands) and 3 µL Annexin V-FITC (Biolegend, Amsterdam, The Netherlands) in Annexin V binding buffer (0.1 M Hepes, 1.4 M NaCl, and 25 mM CaCl2 in deionized water with a pH set to 7.4. sterile filtered using a 0.2 µm filter) and analyzed by flow cytometry on a Cytek Aurora 3-Laser flow cytometer (Cytek, Fremont, CA, USA). As controls, cells were subjected to incubation with 300 pM AU-011 only, 690 nm light only or three freeze/thaw cycles at  − 20 °C (FT) before staining and analysis. For AU-011 PDT in co-culture, 4 × 10^4^ MC38CFP cells were seeded in 24-well plates kept in an incubator overnight. The next morning, an equal number of D1DCs was added to the well after which the co-culture was incubated with 300 pM AU-011 for 4 h at 37 °C. The cells were then washed in PBS, provided with fresh culture medium and illuminated with 690 nm light at 400 mW/cm^2^ for 25, 2.5 or 0.25 J/cm^2^. Cells were then kept in an incubator for 18 h, collected in FACS buffer and stained with anti-CD11c-PE (Clone HL3; BD Biosciences, New Jersey, USA) for 30 min at 4 °C. Cells were then washed in PBS, stained with 3 µL Annexin V-FITC in Annexin V binding buffer for 30 min at 4 °C and washed again in PBS before analysis by flow cytometry on a Cytek Aurora 3-Laser flow cytometer (Cytek, Fremont, CA, USA). As controls, cells were subjected to incubation with 300 pM AU-011 only or laser light only.

### Binding and cytotoxicity comparison in a panel of colon cancer cell lines

This assay has been previously described [16, 27]. Briefly, cells were detached and allowed to recover after which the cells were moved to 96-well round-bottom plates, followed by centrifuged at 1000 RPM. Cells were then resuspended in AU-011 at indicated concentrations and incubated for one-hour at 4 °C. The cells were then washed and resuspended in phenol-red free DMEM (Invitrogen, Waltham, MA, USA) supplemented with 10% FBS. For the comparison of cytotoxicity, half of the cells were transferred to a black, round bottom plate and irradiated with 25 J/cm^2^ of 690 nm light (Modulight ML6700-PDT with MLA kit). The other half of the cells were used for the comparison of binding and the dark toxicity (0 J/cm^2^). Following light treatment, all cells were allowed a 1–2 h recovery at 37 °C. The samples were then stained using LIVE/DEAD Yellow fixable stain (ThermoFisher, Waltham, MA, USA), followed by 10 min fixation in 4% paraformaldehyde and measured by flow cytometry using a BD FACS Canto II outfitted with a high-throughput sampler (BD Biosciences, New Jersey, USA).

### Detection of PDT-induced immunogenic cell death through DAMP exposure and release

The exposure of calreticulin (CRT) and release of HMGB-1 was measured by seeding 5 × 10^4^ MC38 cells, 4 × 10^4^ CT26 cells or 3.5 × 10^4^ TC-1 cells in 24-well plates in culture medium and incubated overnight at 37 °C and 5% CO_2_. Cells were then incubated with 300 pM AU-011 in culture medium for 4 h, and subsequently illuminated with 690 nm light at 400 mW/cm^2^ for 0.25, 2.5, or 25 J/cm^2^. As controls, cells were subjected to incubation with 300 pM AU-011 only, 690 nm light only or three freeze/thaw cycles at  − 20 °C (FT). Samples were then incubated for 18 h, after which the supernatant was collected and frozen at  − 20 °C until further processing to detect the HMGB-1 release by enzyme-linked immunosorbent assay (ELISA). Following collection of the supernatant, the remaining cells were washed three times in PBS, collected in FACS buffer, stained with the antibody anti-calreticulin-FITC (Clone EPR3924; Abcam, Cambridge, UK) and 0.5 µM DAPI in FACS buffer before analysis by flow cytometry on a Cytek Aurora 3-Laser flow cytometer (Cytek, Fremont, CA, USA). The frozen supernatants were thawed after which potentially remaining cells were removed by centrifugation at 300 × g for 5 min. The resulting samples were incubated in 96-well NUNC Maxisorp plates (Thermofisher, Landsmeer, The Netherlands) that were coated overnight with 50 µL of a 5 µg/mL rabbit-anti-HMGB-1 antibody (Novus Biologicals, Centennial, CO, USA) in coating buffer (0.05 M Carbonate-Bicarbonate, pH 9.6) at 4 °C. The plates were then washed three times in washing buffer (PBS with 0.05% Tween 20, pH 8.0) and blocked for 60 min at 37 °C with blocking buffer (PBS with 0.05% Tween and 1% BSA, pH 8.0). The plates were then washed three times in washing buffer and incubated with 150 µL of the samples for 120 min at 37 °C. The plates were subsequently washed three times in washing buffer and incubated with 4 µg/mL mouse-anti human HMG-1/HMGB-1-Biotin (Clone 19N12A1; Novus Biologicals, Centennial, CO, USA) in blocking buffer for 60 min at room temperature. The plates were again washed three times in washing buffer and incubated with Streptavidin-poly-HRP (Thermofisher, Landsmeer, The Netherlands) in blocking buffer for 60 min at room temperature. Plates were then washed five times in washing buffer, dried briefly and incubated with HRP-substrate 3,3',5,5'-tetramethylbenzidine (TMB, Thermofisher, Landsmeer, The Netherlands) until a change of color was clearly visible. Stopping the reaction was achieved by addition of 0.18 M H_2_SO_4_ in deionized water, after which the absorption was measured at 490 nm using a plate reader (Bio-Rad Laboratories, Veenendaal, The Netherlands).

### Maturation of D1DCs post incubation with PDT-treated tumor cells

The immunostimulatory effects of PDT were investigated by seeding 5 × 10^4^ MC38 cells, 4 × 10^4^ CT26 cells or 3.5 × 10^4^ TC-1 cells in 24-well plates and 10^4^ D1DCs in 96-well plates (Corning, Glendale, CA, USA). After overnight incubation at 37 °C and 5% CO_2_, the cancer cells were incubated with 300 pM AU-011 in culture medium for 4 h, and subsequently illuminated with 690 nm light at 400 mW/cm^2^ for 0.25, 2.5, or 25 J/cm^2^. As controls, cells were subjected to incubation with 300 pM AU-011 only, 690 nm light only or three freeze/thaw cycles at  − 20 °C (FT). Following this, the PDT-treated tumor cells were added to the D1DCs at a ratio of 20: 1 (tumor cell: D1DC) and incubated for 24 h at 37 °C and 5% CO_2_. Cells were then collected, stained with 0.5 µM DAPI (Sigma-Aldrich, Zwijndrecht, The Netherlands), CD86-FITC (clone GL1; Thermofisher, Landsmeer, The Netherlands) and anti-CD11c-PE (Clone HL3; BD Biosciences, New Jersey, USA) before analysis by flow cytometry on a Cytek Aurora 3-Laser flow cytometer. D1DC controls consisted of D1DCs in mono-culture, poly I:C at 1 µg/mL, 300 pM AU-011 directly incubated with D1DCs, or 300 pM AU-011 incubated with tumor cells added to D1DCs without treatment (dark toxicity).

### Animals

Male and female C57BL/6-albino mice were bred in the breeding facility of the Leiden University Medical Center (LUMC, Leiden, The Netherlands) and female C57BL/6 J mice were obtained from ENVIGO (Horst, the Netherlands). The animals were housed under specified pathogen-free conditions in the animal facility of the LUMC. The animal experiments were conducted in accordance with the Code of Practice of the Dutch Animal Ethical Commission (animal permit: AVD1160020198405, approved 19 November 2019).

### *Biodistribution of AU-011 *in vivo

To measure the distribution of AU-011 in vivo, C57BL/6-albino mice were subcutaneously inoculated with 5 × 10^5^ MC38 in 200 µL PBS on the right flank. Once the tumors had reached an average volume of approximately 125 mm^3^ as determined by measuring with a caliper, the mice were randomly divided into groups after which 100 µg AU-011 in 100 µL was administered intravenously into the tail vein or intraperitoneally, or 30 µg AU-011 in 30 µL was administered intratumorally. The fluorescence of AU-011 in the tumors was then measured over time using fluorescence spectrometry imaging with the IVIS Spectrum (PerkinElmer, Waltham, MA, USA) under isoflurane anesthesia. Relevant areas were shaved right before measurements to minimize interference by absorption of the fluorescent signal. Measurements were performed at automatic exposure times at position C using filter settings relevant for AU-011. At 96 h post administration of AU-011, mice were sacrificed and the organs were excised for analysis using the IVIS spectrum.

### *AU-011- and Foscan-PDT combined with immune checkpoint inhibition tumor treatment *in vivo

For PDT in vivo, C57BL/6 J mice were inoculated subcutaneously with 5 × 10^5^ MC38 in 200 µL PBS on the right flank. Once the tumors had nearly reached an average volume of approximately 125 mm^3^, the mice were randomly divided into groups and treated with PDT. To this end, AU-011 was administered intravenously into the tail vein at 100 µg in 100 µL dilution buffer. At a drug-to-light interval (DLI) of 12 h, the skin surrounding the tumor area was shaved and tumors were illuminated with 690 nm light under isoflurane anesthesia at a fluence rate of 400 mW/cm^2^ for a total of fluence of 75 J/cm^2^ in 6 pulses of 12.5 J/cm^2^ with 2 min pauses in between. For AU-011 only, or Light only, animals were treated as described above, without light or AU-011, respectively. After PDT treatment, checkpoint blockade antibodies against CTLA-4 (Clone 9H10; BioXCell, Lebanon, PA, USA; 200 µg per administration), PD-L1 (Clone 10F.9G2; BioXCell, Lebanon, PA, USA; 200 µg per administration), LAG-3 (Clone C9B7W; BioXCell, Lebanon, PA, USA; 200 µg per administration) or LAG-3 together with PD-L1 (150 µg of each antibody per administration) were injected intraperitoneally at days 8, 10, 13, and 16 post tumor inoculation. During the entire experiment, the animal condition, weight and tumor volume was measured regularly. For Foscan-PDT (temoporfin obtained from Sigma-Aldrich, Zwijndrecht, The Netherlands), animals were injected at day 6 post inoculation with 0.15 mg/kg Foscan in 30 µL over 4–6 min. At a DLI of 24 h, the skin surrounding the tumor area was shaved and tumors were illuminated with 650 nm light (LED diode laser, Laser2000, Vinkeveen, The Netherlands) under isoflurane anesthesia at a fluence rate of 30 mW/cm^2^ for a total fluence of 50 J/cm^2^.

### Induction of an abscopal effect after AU-011 PDT combined with immune checkpoint inhibition

C57BL/6 J mice were inoculated subcutaneously with 5 × 10^5^ MC38 in 200 µL PBS on the right flank (designated ‘primary tumor’) and with 2.5 × 10^5^ MC38 in 200 µL PBS on the left flank (designated ‘distant tumor’). Once the primary tumors had reached an average volume of approximately 125 mm^3^, the mice were randomly divided into groups, after which the primary tumor (on the right flank) was treated with PDT as described. Subsequently, checkpoint blockade antibodies against CTLA-4 (Clone 9H10; BioXCell, Lebanon, PA, USA; 200 µg per administration), PD-L1 (Clone 10F.9G2; BioXCell, Lebanon, PA, USA; 200 µg per administration), LAG-3 (Clone C9B7W; BioXCell, Lebanon, PA, USA; 200 µg per administration) or LAG-3 together with PD-L1 (150 µg of each antibody per administration) were injected intraperitoneally at days 8, 10, 13, and 16 post inoculation. During the entire experiment, the animal’s condition, weight and tumor volume was measured regularly.

### Statistics

Graph Pad Prism software version 9 was used for statistical analysis, FlowJo was used for flow cytometry data and Living Image was used for processing biodistribution in vivo data obtained with the IVIS Spectrum. Data were analyzed as indicated for individual experiments.

## Results

### AU-011 preferentially associates with cancer over dendritic cells

**Fig. 1:**
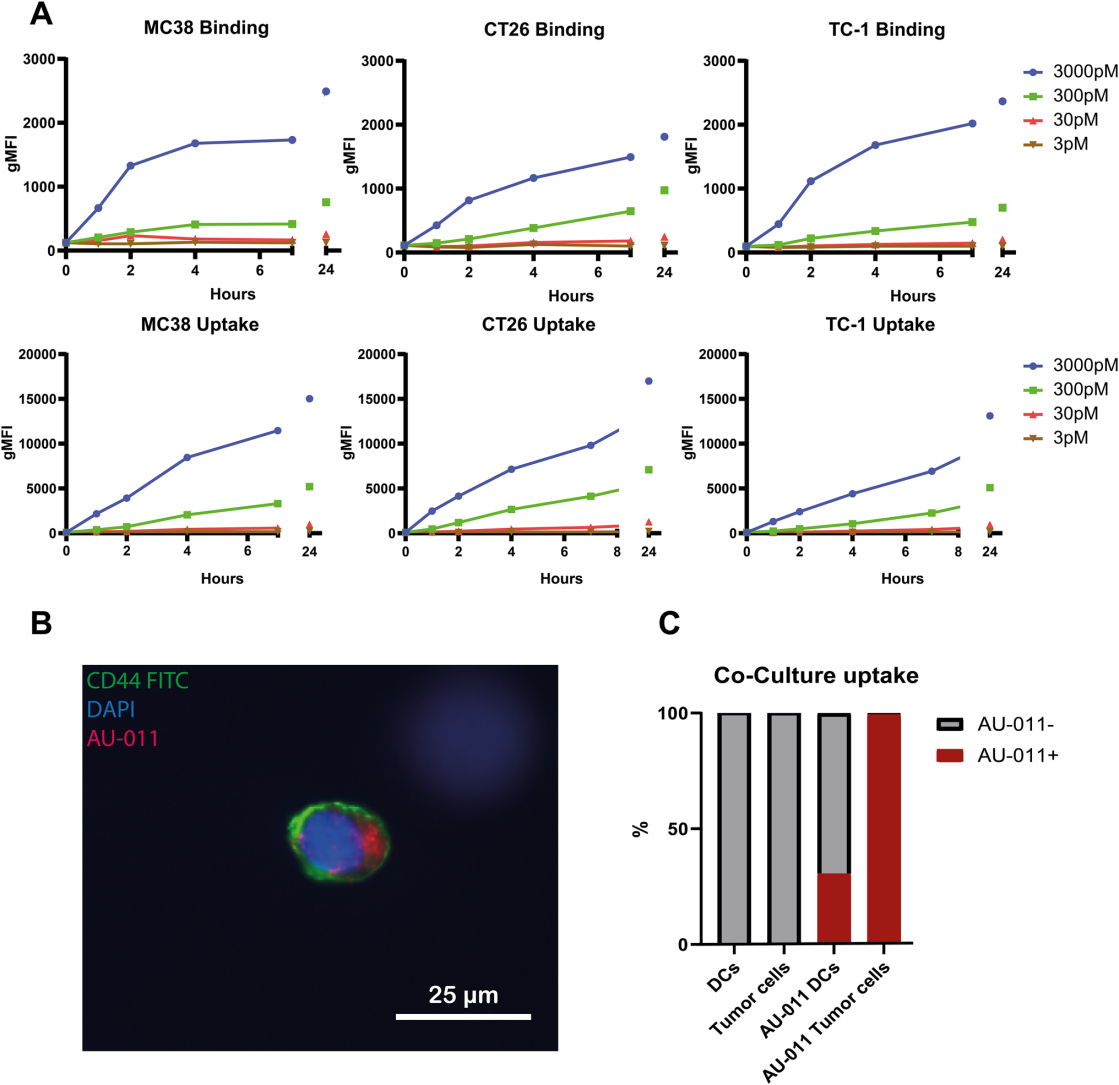
Internalization and binding of AU-011 in cancer and APCs. **A** Murine cell lines MC38, CT26, and TC-1 were incubated with 3–3000 pM of AU-011 in the dark at 4 °C (binding) or 37 °C (uptake) for 0–24 h. The fluorescence of AU-011 was then measured by flow cytometry. **B** MC38 cells were seeded in 8-chamber polystyrene slides with removable wells and incubated with 300 pM AU-011 for 4 h at 37 °C. Cells were stained with CD44-FITC (membrane staining) and coverslips were mounted in Vectashield mounting medium containing DAPI (nucleus staining). Slides were analyzed by fluorescence microscopy. **C** An equal number of MC38CFP and D1DCs was incubated with AU-011 for 4 h at 37 °C. Cells were then stained with CD11c-PE, after which the fluorescence of AU-011 was measured using flow cytometry. Separation of MC38CFP and D1DCs was based on CFP^+^ (cancer cells) or CD11c^+^ (immune cells). The y-axis indicates the percentage of cells positive for AU-011. Data are representative of three separate experiments

We first set out to investigate whether AU-011 bound to and was internalized in murine colon cancer cell lines Murine Colon 38 (MC38) and CT26, in addition to the HPV16 E6 and E7-expressing murine tumor cell line TC-1 to which binding was previously shown [27]. For this, cells were incubated with several concentrations of AU-011 for an indicated time at 4 °C (Fig. 1a, upper panels), to measure AU-011 binding to cancer cells, or at 37 °C (Fig. 1a, lower panels), to measure the total amount of AU-011 associated with cancer cells through binding and internalization. Analysis by flow cytometry showed that the total amount of AU-011 binding increased over time for all cell lines, whereby the levels increased with rising concentrations (Fig. 1a, upper panels). AU-011 uptake similarly increased over time for all tested murine cancer cell lines after incubation at 37 °C (Fig. 1a, lower panels). However, the mean fluorescence intensity for binding only (Fig. 1a, upper panels) was much lower compared to binding and internalization combined (Fig. 1a, lower panels). Moving forward, all incubations were performed at 37 °C unless indicated otherwise. Internalization of AU-011 was confirmed by fluorescence microscopy, showing the fluorescence of AU-011 in the cytoplasm (Fig. 1b). To determine whether AU-011 preferentially associates with cancer over APCs, D1 Dendritic Cells (D1DCs) were co-incubated with Cyan Fluorescent Protein (CFP)-expressing MC38 cells (MC38CFP) at an equal number of cells. The co-culture was then incubated with 300 pM of AU-011 for 4 h at 37 °C and analyzed by flow cytometry. Approximately 30% of D1DCs were found to be positive for AU-011 versus approximately 100% of tumor cells in co-culture (Fig. 1c), indicating that AU-011 displays a measure of preference for cancer over APCs in co-culture.

### AU-011 treatment with light activation preferentially kills cancer cells over APCs

**Fig. 2:**
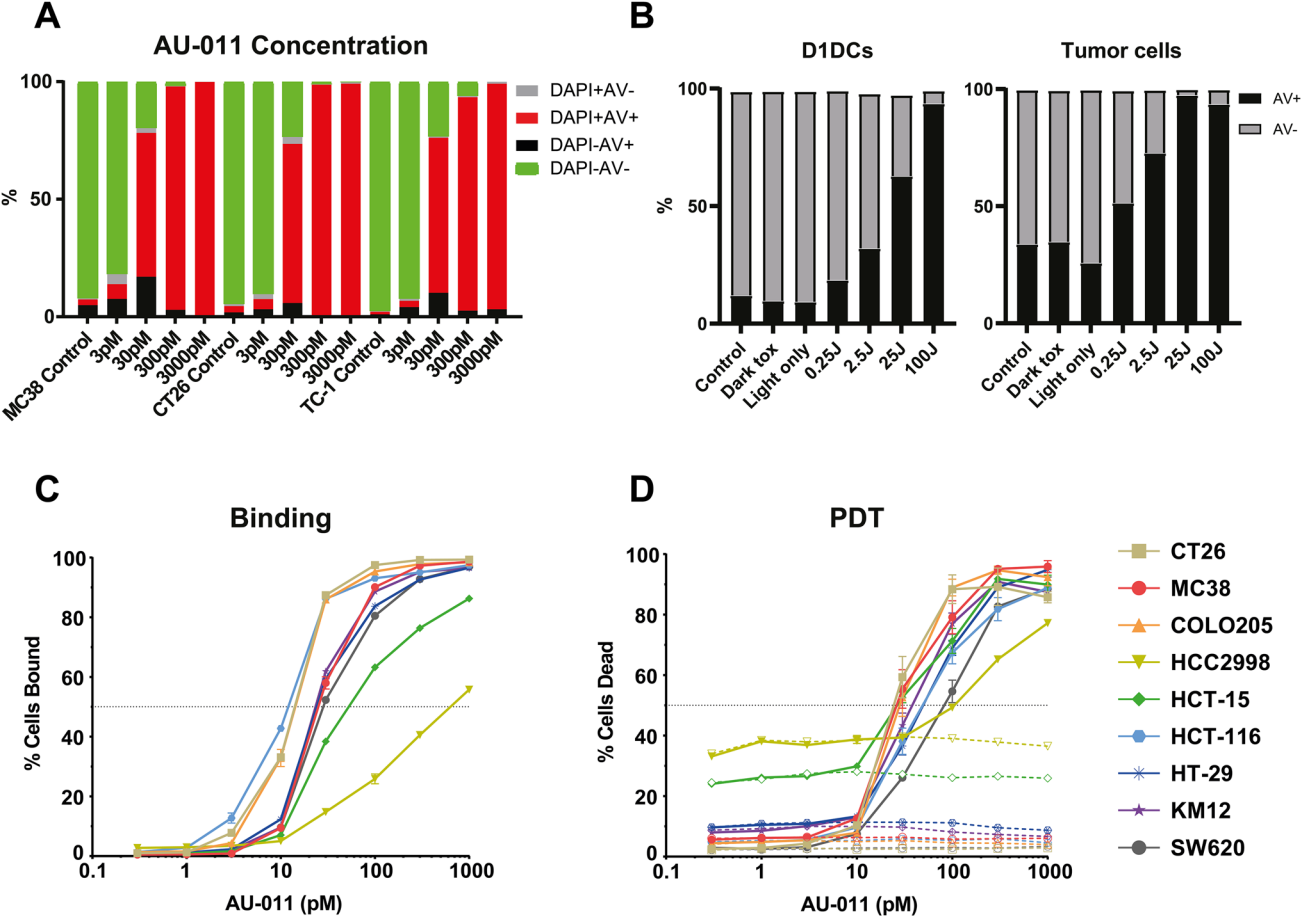
AU-011 treatment with light activation preferentially induces cytotoxicity in cancer cells over APCs. **A** MC38, CT26 or TC-1 were incubated with 3–3000 pM of AU-011 in the dark for 4 h. Cells were then illuminated with NIR light (690 nm) light at 400 mW/cm^2^ for 25 J/cm^2^. At 18 h after treatment, the samples were stained with viability markers Annexin V-FITC and DAPI before analysis by flow cytometry. Representative graph of ≥ 3 independent experiments. **B** A co-culture containing an equal number of MC38CFP and D1DCs were incubated with 300 pM of AU-011 in the dark for 4 h. Cells were left in the dark (dark toxicity, dT), treated with NIR light only at 400 mW/cm^2^ for 100 J/cm^2^ (light only), treated with AU-011 with light activation by illumination with NIR at 400 mW/cm^2^ for 0.25–100 J/cm^2^ or not treated with either AU-011 or light (control). At 18 h after treatment, the samples were stained with CD11c-PE and viability marker Annexin V-FITC before analysis by flow cytometry. Separation of MC38CFP and D1DCs was based on CFP^+^ (cancer cells) or CD11c^+^ (immune cells), showing (left panel) D1DCs and (right panel) tumor cells. A panel of colon cancer cell lines was tested for **C** percentage of cells bound to AU-011 and **D** cytotoxicity of AU-011 PDT at various concentrations of PS, comparing dark toxicity (dotted lines) with PDT treatment at 25 J/cm.^2^ (solid lines). (mean ± SEM; n = 3)

The capacity of AU-011 treatment with light activation to induce cytotoxicity in MC38, CT26, and TC-1 cells was investigated. To this end, cells were incubated with AU-011 for 4 h, washed and subsequently illuminated with near-infrared (NIR) light (690 nm). Cells were then incubated for 18 h, stained with early-apoptotic marker Annexin V-FITC and death marker 4′,6-diamidino-2-phenylindole (DAPI) before analysis by flow cytometry. The effects of light only and of AU-011 in the absence of light (dark toxicity) were investigated, showing no or negligible toxicity even at high concentrations of AU-011 (Fig. S1a, b). For AU-011, cells were incubated with varying concentrations of AU-011 for 4 h and subsequently illuminated with NIR light (690 nm) at 400 mW/cm^2^ for 25 J/cm^2^ (Fig. 2a). Complete or near-complete cell death was observed after AU-011 for all cell lines treated with 3000 pM and 300 pM, followed by approximately 75% cell death observed for PDT with 30 pM, and below 15% cell death observed after PDT with 3 pM. Similar results were obtained when varying fluence instead of AU-011 concentration (Fig. S1c), showing near-complete cell death for fluences upward from 2.5 J/cm^2^, while varying fluence rate did not have an effect on cytotoxicity (Fig. S1d). There results indicate that the in vitro cytotoxicity of AU-011 is dependent on the concentration of AU-011 and the fluence, but not the fluence rate used for light activation.

To test whether AU-011 preferentially kills cancer cells over APCs, D1DCs were co-cultured with MC38CFP cells and incubated with 300 pM AU-011 for 4 h before illumination at 400 mW/cm^2^ for 25 J/cm^2^. The dark toxicity and light-only incubations did not induce a significant difference from untreated samples (control) for both D1DCs (Fig. 2b, left panel) and tumor cells (Fig. 2b, right panel). However, approximately 25–30% of tumor cells in co-culture stained positive for early-apoptotic marker Annexin V for the control, dark toxicity and light-only conditions (Fig. 2d), indicating that the viability of tumor cells is affected in a co-culture with D1DCs, regardless of the presence of AU-011. Near-complete cell death is induced after PDT at 100 J/cm^2^ in both D1DCs (Fig. 2c) and tumor cells (Fig. 2d). However, only approximately 60% of D1DCs versus 96% of tumor cells are positive for Annexin V at 25 J/cm^2^, 30% of D1DCs versus 75% of tumor cells at 2.5 J/cm^2^, and 15% of D1DCs versus 50% of tumor cells at 0.25 J/cm^2^. These results show that AU-011-mediated cytotoxicity is preferentially induced in cancer cells versus APCs in a co-culture. As tumors consist of a mix of immune and cancer cells, these results indicate the potential for a preference of the treatment for cancer cells over APCs in vivo.

To test whether there is AU-011 related homogeneity between different colon cancers, we compared the binding (Fig. 2c) and cytotoxicity (Fig. 2d) in a panel of murine and human colon cancer cell lines. The results show an increase in the percentage of AU-011-bound cells for all cell lines tested (Fig. 2c). All cell lines displayed an excellent dark toxicity at all AU-011 concentrations included (Fig. 2d). Although the baseline cytotoxicity for HCT-15 and HCC2998 was relatively high compared to the other lines, this cytotoxicity did not increase upon incubation with AU-011 in the dark (Fig. 2d), underlining the excellent dark toxicity of AU-011. For most cell lines, AU-011 with light activation induced cytotoxicity and this effect was increased with drug concentration, inducing near-complete cell death starting at a concentration of 300 pM. This effect was slightly reduced in HCC2998 cells that displayed a lower percentage of cell death after treatment in spite of a relatively high baseline cytotoxicity (Fig. 2d). This may be related to the reduced binding capacity of AU-011 to these cells (Fig. 2c), resulting in reduced cell death. These results indicate that most, but not all colon cancer cell lines behave similarly in terms of binding capacity to AU-011 and cytotoxicity after light activation of AU-011- in vitro.

### Treatment with AU-011 induces immunogenic cell death and dendritic cell maturation

**Fig. 3:**
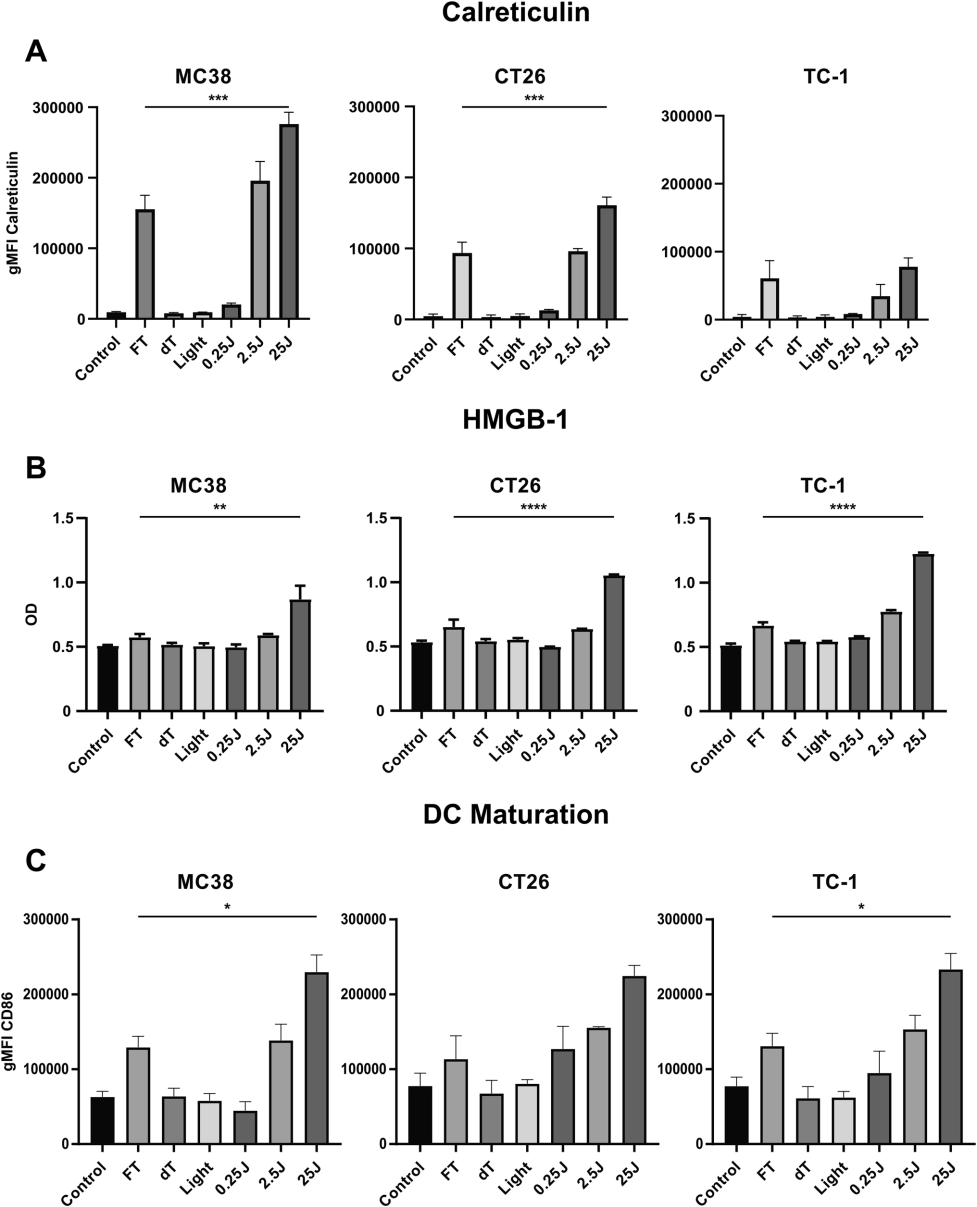
AU-011 treatment with light activation induces exposure and release of DAMPs in addition to maturation of D1DCs. **A** MC38, CT26, and TC-1 were incubated with 300 pM of AU-011 in the dark for 4 h. Cells were then illuminated with NIR light (690 nm) at 400 mW/cm^2^ for 0.25–25 J/cm^2^. Controls were left untreated (control), subjected to three cycles of freeze-thawing at  − 20 °C (FT), treated with 300 pM AU-011 only (dark toxicity, dT) or treated with NIR light at 400 mW/cm^2^ for 25 J/cm^2^ only (Light). At 18 h after treatment, the samples were labeled with anti-calreticulin antibodies before analysis by flow cytometry. Flow cytometry data are displayed as the geometric mean fluorescence intensity (gMFI). **B** Supernatants were collected at 18 h after treatment as in (**A**) and analyzed for the presence of HMGB-1 by ELISA, displayed as optical density (OD). **C** Samples treated as in (**A**) were incubated with D1DCs for 24 h immediately after treatment. Samples were then stained with DAPI, anti-CD11c-PE and anti-CD86-FITC before analysis by flow cytometry. Gating of living D1DCs was based on DAPI^−^CD11c^+^ events and CD86 levels are displayed as the gMFI of anti-CD86-FITC. Statistical analysis was performed using a one-way ANOVA with Tukey correction for multiple comparisons (*p < 0.05, **p < 0.01, ***p < 0.001, ****p < 0.0001; mean ± SD; n = 3)

Photodynamic therapy using photosensitizers like the chlorin e6-based Radachlorin has been shown to induce immunogenic cell death, through exposure and release of DAMPs [32, 33]. Moreover, PDT can disrupt the structural integrity of cancer cells, theoretically exposing previously inaccessible (neo-)epitopes in the tumor that can be phagocytosed, processed and presented by professional antigen-presenting cells (APCs) in the inflammatory environment, possibly inducing tumor-specific T cell responses. In line with this, we have previously shown that PDT using Radachlorin combined with nanoparticle-encapsulated immunostimulatory agents initiates tumor (neo)epitope- and tumor-specific T cell responses, and induces partial responses in multiple murine tumor models [34]. Here, we investigated the ability of AU-011 to induce immunogenic cell death through the release and exposure of DAMPs. To this end, cells were incubated with 300 pM AU-011 for 4 h followed by illumination with NIR light (690 nm) at 400 mW/cm^2^ for 25 J/cm^2^. For the detection of calreticulin (CRT) exposure after light activation, cells were collected and analyzed by flow cytometry at 18 h after treatment. The results show a strong increase in CRT exposure after PDT, increasing with higher fluence to a level exceeding three cycles of freeze/thawing (FT) at  − 20 °C (Fig. 3a). This effect is most pronounced in MC38 cells, followed by CT26 cells, as measured at 18 h after treatment, and indicates that light activation induces upregulation of CRT before cell death. The smallest increase in CRT exposure is observed in TC-1 cells, where the levels of CRT exposure did not exceed the level after three cycles of FT at  − 20 °C (Fig. 3a). The light activation-induced release of HMGB-1 was determined by collection of the supernatant at 18 h after treatment, followed by analysis using enzyme-linked immunosorbent assay (ELISA). The results show a strong HMGB-1 release that increases with higher fluence in TC-1 cells and to a lesser extent in CT26 cells, followed by a slight increase in HMGB-1 release in MC38 cells (Fig. 3b). In all cell lines, the level of HMGB-1 release is increased compared to three cycles of FT, indicating that HMGB-1 expression is also increased after AU-011 treatment. These results show that AU-011 treatment, at a fluence that induces near-complete cell death, facilitates the exposure and release of DAMPs.

To test whether AU-011-induced DAMP exposure and release from dying cancer cells is able to result in the maturation of dendritic cells, cancer cells were treated with AU-011 as described and then co-cultured with D1DCs for 24 h. The expression of the maturation marker CD86 on living (DAPI^−^) D1DCs was evaluated by flow cytometry (Fig. 3c). For all cell lines, the levels of maturation marker CD86 increased with fluence to a level that exceeds all controls including three cycles of FT and incubation with 1 µg/mL of the toll-like receptor ligand poly I:C (Fig. S2). Moreover, the levels of CD86 expression in D1DCs after AU-011 was comparable between cell lines, with the exception of 0.25 J/cm^2^ for MC38 that displays a slightly lower expression level compared to D1DCs incubated with CT26 and TC-1. These results show that MC38 and TC-1 display the highest and lowest CRT cell surface levels and the lowest and highest HMGB-1 release, respectively, with CT26 in the middle in both cases. However, all three AU-011-treated cancer cell lines induce DC maturation, with similar levels of maturation marker expression, in spite of these differences in DAMP exposure and release.

### Biodistribution of AU-011 in a colon tumor-bearing murine model

**Fig. 4:**
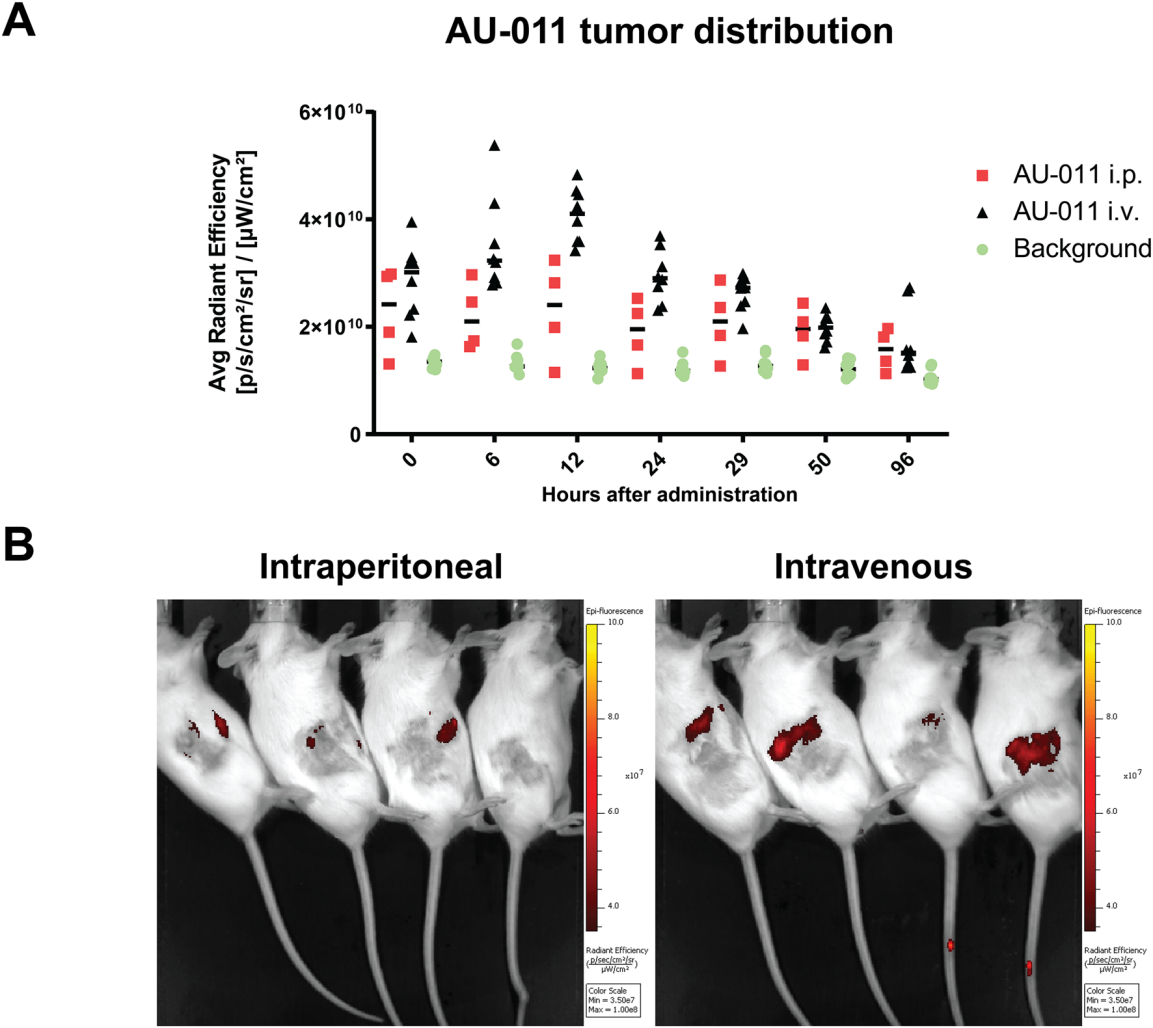
Biodistribution of AU-011 after administration in murine models. **A** Male and female C57BL/6-albino mice, randomly divided among groups, were inoculated with 0.5 × 10^6^ MC38 cells in the right flank. At 7 days post inoculation, when tumors were established (125 mm^3^), the mice were injected with 100 µg of AU-011 intraperitoneally (i.p.) or intravenously (i.v.) into the tail vein. The fluorescence of AU-011 was measured in the tumors over time on the IVIS Spectrum fluorescence spectrometer and compared to untreated mice bearing MC38 tumors (background). **B** Representative IVIS images of animals injected intraperitoneally or intravenously with AU-011 at 12 h post administration. (mean; n = 4–7)

Expanding on the preferential in vitro cytotoxicity for cancer cells over APCs and the ability of AU-011 treatment to induce DAMP exposure in addition to dendritic cell maturation, we investigated the distribution of AU-011 in MC38 tumor-bearing mice. To test this, C57BL/6 J albino mice were inoculated subcutaneously with 5 × 10^5^ MC38 cells in the right flank. When the tumors were established (~ 125mm^3^), the mice were randomly divided among groups and injected with AU-011 intraperitoneally, intravenously into the tail vein, or intratumorally. After this, the fluorescence of AU-011 was measured in the tumors over time by *in* vivo fluorescence spectrometry (Fig. 4a, b). The average radiance efficiency of AU-011 in the tumors increased up to 12 h post intravenous administration and displayed reduced variation between the animals with the lowest and highest radiance compared to 6 h (Fig. 4a, b). Signal in the tumor was observed up to 96 h post administration, after which the fluorescence decreased to near-background levels. For intraperitoneal injection, the signal was increased compared to the background and remained relatively constant over time (Fig. S3a, Fig. 4a, b). However, the tumor fluorescence was lower compared to intravenous injection at 12 h post administration. As a control, intratumoral injection displayed very strong tumor fluorescence that was slowly reduced over time (Fig. S3b, c). In addition to the tumor, AU-011 distributed to the tumor-draining lymph nodes, spleen, kidney, lung and eye (Fig. S3d) at 96 h post administration, showing distribution to various organs throughout the body. These results show that AU-011 accumulates in tumors over time after intravenous administration, with a peak and low signal spread around 12 h post administration.

### Treatment with AU-011 treatment combined with immune checkpoint inhibition induces complete and durable responses in a subcutaneous murine tumor model

To test the efficacy of AU-011 treatment in vivo, C57BL/6 J mice were inoculated subcutaneously with 0.5 × 10^6^ MC38 cells in the right flank. When the tumors were established (~ 125mm^3^), the animals were injected intravenously with 100 µg AU-011 in the tail vein. Based on the results of the biodistribution, a drug-to-light-interval (DLI) of 12 h post intravenous injection was chosen after which the tumors were illuminated with NIR light (690 nm) at 400 mW/cm^2^ for 50 J/cm^2^ in four pulses of 12.5 J/cm^2^ at 2 min intervals, 75 J/cm^2^ in six pulses of 12.5 J/cm^2^ at 2 min intervals, and 100 J/cm^2^ in eight pulses of 12.5 J/cm^2^ at 2 min intervals (Fig. S4a). A fluence of 75 J/cm^2^ induced an optimal tumor growth inhibition (Fig. S4b), without inducing a difference in animal weight (Fig. S4c), and was therefore chosen as the fluence for further experiments. Using these conditions, AU-011 treatment was compared to the EMA-approved PDT drug, Foscan (Fig. S5a). The effect of AU-011 only and NIR light only (690 nm, 400 mW/cm^2^ for 75 J/cm^2^) on MC38 tumor growth was comparable to no treatment (Fig. S5c, d; Fig S6). Again, no difference in animal weight was observed between the groups (Fig. S5b). The tumor growth inhibition of AU-011 treatment was comparable to PDT with Foscan (Fig. S5c, d; Fig. S6 (individual growth curves)), showing the potency of the optimized AU-011 treatment protocol when compared to clinically-approved sensitizers.

**Fig. 5:**
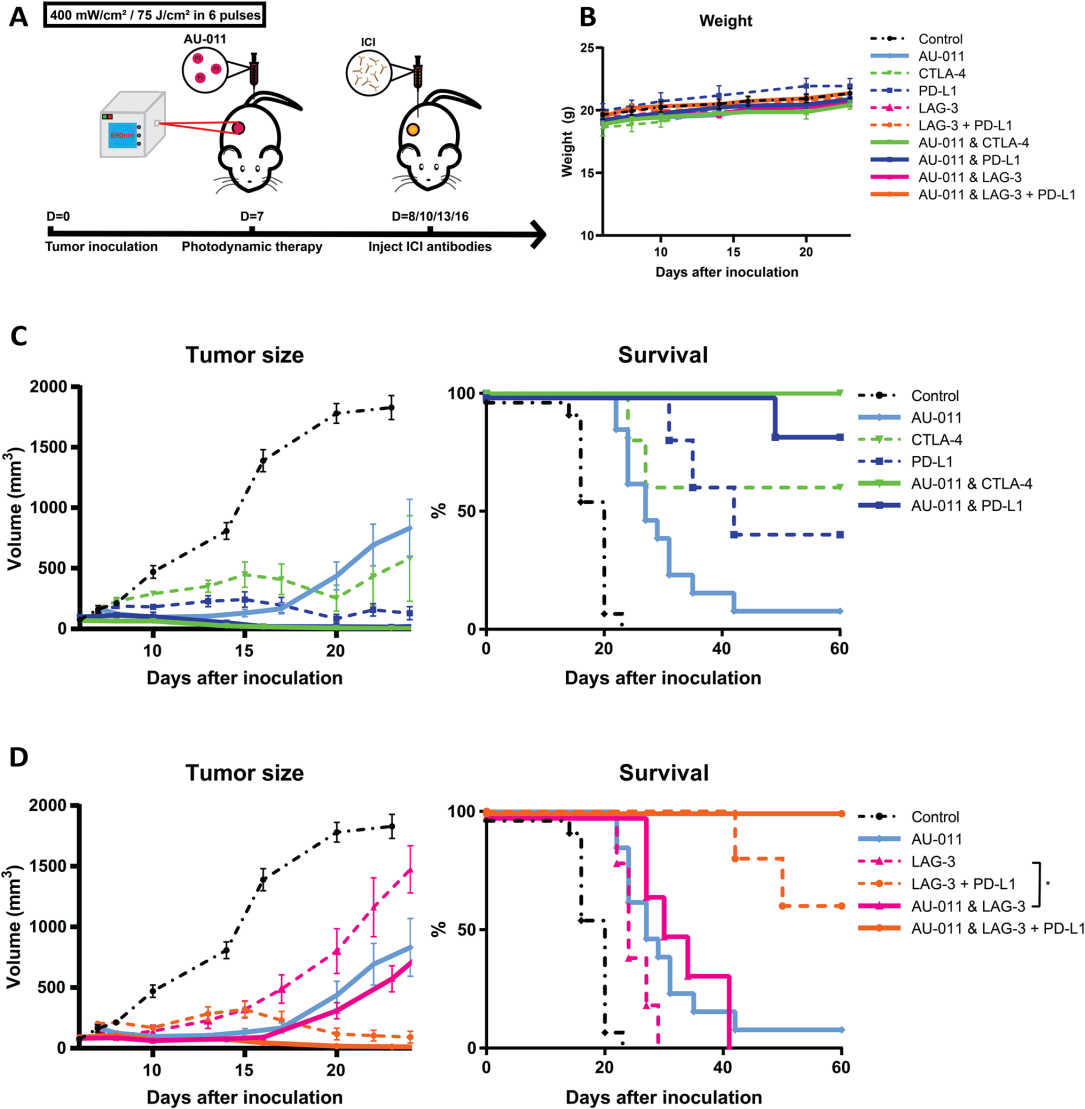
AU-011 as a single agent and in combination with immune checkpoint inhibition in murine tumor models. **A** C57BL/6 mice were inoculated with 0.5 × 10^6^ MC38 cells in the right flank. At 7 days post inoculation, when tumors were established (125 mm^3^), the mice were injected with 100 µg of AU-011 intravenously into the tail vein. The tumors were then illuminated with NIR light (690 nm) at a DLI of 12 h with 400 mW/cm^2^ for 75 J/cm^2^. At days 8, 10, 13 and 16 post inoculation, immune checkpoint inhibitory antibodies CTLA-4 (200 µg per administration), PD-L1 (200 µg per administration), LAG-3 (200 µg per administration) or LAG-3 together with PD-L1 (150 µg of each antibody per administration) were injected intraperitoneally, after which the animals were monitored over time. **B** Animal weight of all groups. **C** Tumor volume and survival curves of the animals treated with CTLA-4, PD-L1, PDT with CTLA-4 and PDT with PD-L1. **D** Tumor volume and survival curves of the animals treated with LAG-3, LAG-3 + PD-L1, PDT with LAG-3 and PDT with LAG-3 + PD-L1. Statistical analysis was performed using the ANOVA at day 20 post inoculation for comparison of tumor volume and the Mantel-Cox test for a comparison of survival. (*p < 0.05; mean ± SEM; n = 7–12)

To determine an immunotherapeutic candidate for the combination with AU-011 treatment, checkpoint blockade antibodies anti-CTLA-4, anti-PD-L1, anti-LAG-3 and anti-PD-L1 with anti-LAG-3 [28], administered intraperitoneally, alone or in combination with AU-011 PDT using the optimized protocol beginning one day after AU-011 treatment (Fig. 5a). The treatments did not induce a difference in weight compared to controls (Fig. 5b), indicating that the treatments were well tolerated. All treatments inhibited tumor growth compared to control (Fig. 5c, d; Fig. S6 (individual growth curves); Table 1) at the day 20 after inoculation, the timepoint when most control animals reached their human endpoints, and led to a notable increase in survival (Fig. 5c, d). Complete responses (CR), defined in this study as animals that have cleared their tumor(s) and remained tumor-free until the end of the experiment, were observed for all animals receiving PDT treatment combined with either anti-CTLA-4 (Fig. 5c) or with anti-PD-L1 together with anti-LAG-3 (Fig. 5d), with responses lasting up to 60 days post-inoculation. The combination of PIT with anti-PD-L1 also strongly inhibited tumor growth (Fig. 5c), with CR in > 80% of animals. However, PDT alone and PDT with anti-LAG-3 displayed a much weaker tumor growth inhibition (Fig. 5d), with only one or no CR after treatment, respectively. Finally, the inhibitory antibodies without PDT also strongly inhibited tumor growth with several CR in 40–60% of cases (Fig. 5c, d), with the exception of anti-LAG-3 alone which did not induce cures, but did inhibit tumor growth compared to controls (Fig. 5d). This high efficacy of the inhibitory abtibodies as a monotreatment, results in a small window for significant improvement by combination AU-011 treatment. In line with this, only anti-LAG-3 combined with PDT showed statistical significance compared to anti-LAG-3 alone. These results show that PDT combined with the checkpoint blockade antibodies anti-CTLA-4, anti-PD-L1 as well as anti-LAG-3 and anti-PD-L1, induce complete and lasting responses against MC38 tumors in this murine model. The antibodies anti-CTLA-4, anti-PD-L1 and anti-LAG-3 with anti-PD-L1 also strongly inhibited tumor growth and enhanced survival in the absence of PDT, albeit to a lesser extent.

**Table 1:** Significance of the data presented in Fig. 5, determined by a one-way ANOVA with Tukey correction for multiple comparisons at day 20 post inoculation for tumor volume and a Mantel-Cox test for survival (*p < 0.05, **p < 0.01, ***p < 0.001, ****p < 0.0001; n ≥ 8)

		AU-011	CTLA-4	PD-L1	LAG-3	LAG-3 + PD-L1	AU-011 & CTLA-4	AU-011 & PD-L1	AU-011 & LAG-3	AU-011 & LAG-3 + PD-L1
Control	Tumor volume	****	****	****	****	****	****	****	****	****
	Survival	****	****	****	***	****	****	****	****	****
AU-011	Tumor volume	–	ns	ns	ns	ns	*	ns	ns	ns
	Survival	–	ns	*	ns	**	***	**	ns	***

### AU-011 treatment combined with immune checkpoint inhibition is effective in a murine model of local and distant tumors

**Fig. 6:**
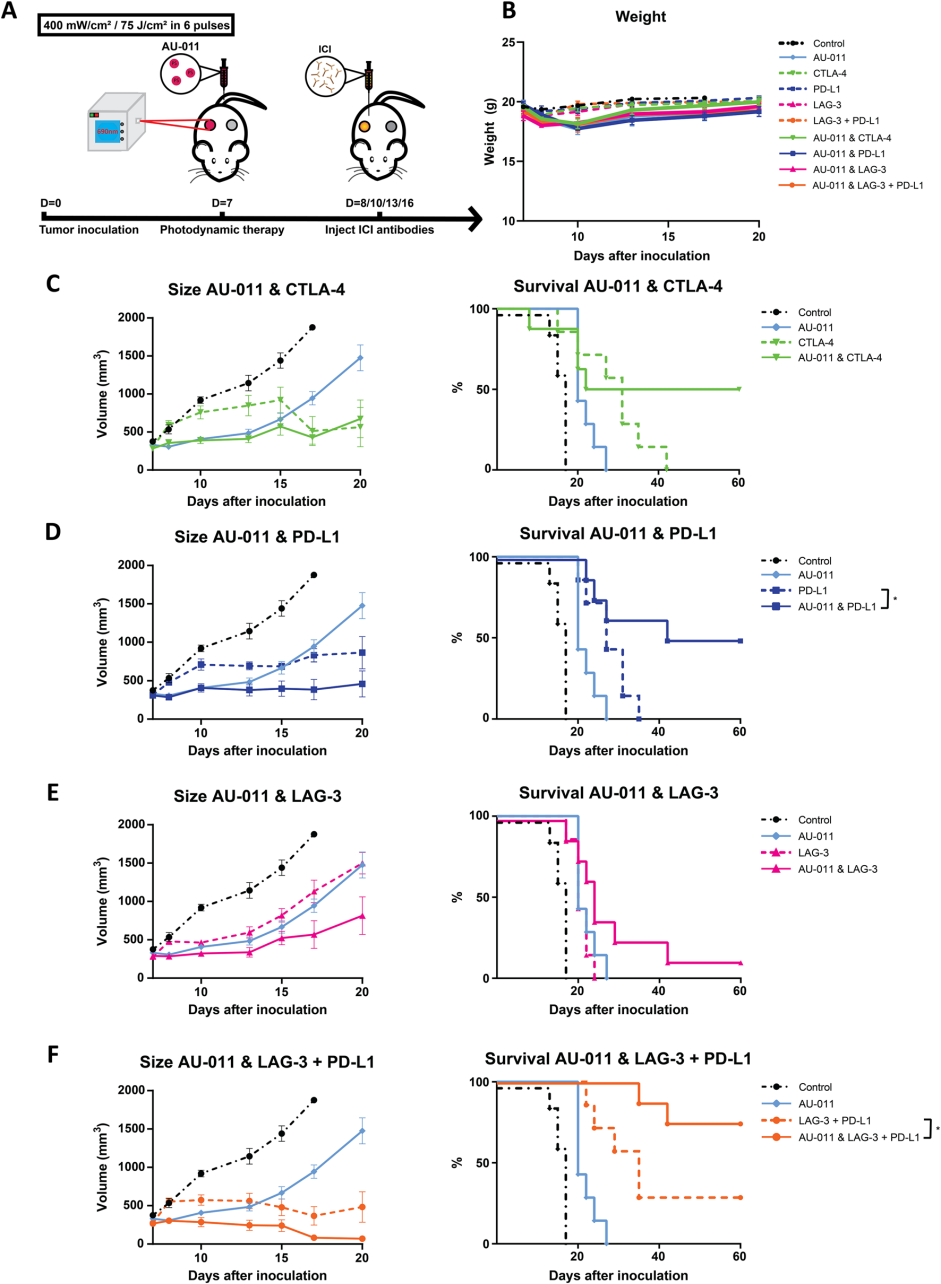
AU-011 treatment enhances immune checkpoint inhibition in primary and distant tumors. **A** C57BL/6 mice were inoculated with 0.5 × 10^6^ MC38 cells in the right flank (primary tumor injection site) and 0.25 × 10^6^ MC38 cells in the left flank (distant tumor injection site). At 7 days post inoculation, when tumors were established (125 mm^3^), the mice were injected with 100 µg of AU-011 intravenously into the tail vein. The primary tumors on the right flank were then illuminated with NIR light (690 nm) at a DLI of 12 h with 400 mW/cm^2^ for 75 J/cm^2^. At days 8, 10, 13 and 16 post inoculation, immune checkpoint inhibitory antibodies CTLA-4 (200 µg per administration), PD-L1 (200 µg per administration), LAG-3 (200 µg per administration) or LAG-3 together with PD-L1 (150 µg of each antibody per administration) were injected intraperitoneally, after which the animals were monitored over time and compared to control (without AU-011, light and immune checkpoint inhibitory antibodies). **B** Animal weight of animals corresponding to the protocol as described. The total tumor burden (cumulative of the primary and distant tumors) and survival curves of animals treated with **C** AU-011 and CTLA-4, **D** AU-011 and PD-L1, **E** AU-011 and LAG-3 and **F** AU-011 and LAG-3 with PD-L1, corresponding to the protocol as described. Statistical analysis was performed using the Mantel-Cox test. (*p < 0.05; mean ± SEM; n ≥ 8)

Expanding on the finding that combining AU-011 with checkpoint blockade antibodies induces a robust antitumor efficacy, we investigated whether the treatment would also be effective against distant and untreated tumors, as a model for metastatic tumors. To this end, C57BL/6 J mice were inoculated subcutaneously with 0.5 × 10^6^ MC38 cells in the right flank, designated ‘primary tumors’, and 2.5 × 10^5^ MC38 cells in the left flank, designated ‘distant tumors’ (Fig. 6a). When the primary tumors (on the right flank) were established (~ 125mm^3^), they were treated with AU-011 as described while the distant tumors (on the left flank) were left untreated. The checkpoint blockade antibodies were injected intraperitoneally one day following AU-011 treatment and the tumor growth was subsequently followed over time (Fig. 6a). Again, the weight of the treated animals was not subtantially different from untreated animals (control), indicating acceptable treatment-induced toxicity (Fig. 6b). Similar to the unilateral tumor model, all treatments significantly inhibited tumor growth at the day 17 after tumor inoculation, the timepoint when control animals reached their human endpoints, and enhanced survival compared to control (Table 2). The AU-011 treatments, either alone or combined with checkpoint blockade antibodies, induced a pronounced effect on the primary tumors at early timepoints after treatment [Fig. S7 (individual growth curves), Fig. S8a (average growth curves)]. AU-011 treatment of the primary tumor alone did not lead to significant tumor growth inhibition of the distant (untreated) tumors [Fig. S7 (individual growth curves), Fig. S8b (average growth curves)], however, the total tumor burden was reduced compared to control as a result of its effect on the primary tumors (Fig. S8c). Of the checkpoint blockade antibodies, anti-CTLA-4 applied as monotreatment induced a tumor growth delay on the primary as well as the distant tumor and, consequently, on the total tumor burden that outperfomed AU-011 as a monotreatment (Fig. 6c). When combined with AU-011 treatment, CR were observed in 50% of animals (Fig. 6c). Although the survival increase for the combination AU-011 and anti-CTLA-4 was not statistically significant, no cures were for anti-CTLA-4 as monotreatment. Similar to anti-CTLA-4, anti-PD-L1 as a monotreatment induced a tumor growth delay without inducing cures (Fig. 6d), but resulted in CR in 50% of animals when combined with AU-011 (Fig. 6d). Conversely, anti-LAG-3 alone showed comparable tumor growth inhibition to AU-011 alone (Fig. 6e). Moreover, AU-011 only slightly improved the survival of animals treated with anti-LAG-3 (Fig. 6e), with approximately 15% of animals cured for AU-011 with anti-LAG-3. Combining anti-LAG-3 and anti-PD-L1 induced CR lasting up to at least 60 days post inoculation in approximately 30% of animals (Fig. 6f). The combination of AU-011 with anti-LAG-3 and PD-L1 induced the strongest tumor growth inhibition and significantly enhanced survival, resulting in CR in approximately 75% of animals at 60 days post-inoculation (Fig. 6f). Overall, the combination of AU-011 with checkpoint blockade antibodies improved upon AU-011 efficacy by consistently showing enhanced tumor growth inhibition as demonstrated by the total tumor burden (Fig. S8c) and increased survival (Fig. S8d) regardless of the antibody used. In addition, the results show that AU-011 combined with anti-CTLA-4, PD-L1 and LAG-3 with PD-L1, but not LAG-3 alone, is more effective in reducing the total tumor burden and enhancing the survival of animals bearing bilateral tumors than either treatment alone.

**Table 2:** Significance of the data presented in Fig. 6, determined by a one-way ANOVA with Tukey correction for multiple comparisons at day 17 post inoculation for tumor volume and a Mantel-Cox test for survival (*p < 0.05, **p < 0.01, ***p < 0.001, ****p < 0.0001; n ≥ 8)

		AU-011	CTLA-4	PD-L1	LAG-3	LAG-3 + PD-L1	AU-011 & CTLA-4	AU-011 & PD-L1	AU-011 & LAG-3	AU-011 & LAG-3 + PD-L1
Control	Tumor volume	***	****	****	**	****	****	****	****	****
	Survival	***	**	***	**	***	**	***	***	***
AU-011	Tumor volume	–	ns	ns	ns	*	ns	*	ns	***
	Survival	–	*	*	ns	**	ns	**	ns	****

## Discussion

Metastasis remains the leading cause of cancer mortality and morbidity, and it is estimated to account for approximately > 66% of all cancer-related deaths [17, 18]. Although photodynamic therapy with photosensitizers is a modality that has shown anti-tumor activity, it is often difficult to treat distant tumors due to the inability of treatment light to reach the location of these tumors, either due to limited penetration depth of the light or inability to insert fiber optics proximal to the desired site of action. We propose a new therapeutic approach that involves treatment of the primary tumor with a novel virus like drug conjugate (AU-011) in combination with immunotherapy to enable a robust effect not only to the primary but also to distant tumors. AU-011 is a novel targeted therapy that is activated with near infrared light using the same laser system that has been traditionally used in photodynamic therapy and that is used to activate other photosensitizers. Upon light activation, the goal of the treatment with AU-011 is to target and selectively kill cancer cells present in the primary tumor with a pro-immunogenic cell death that results in a durable antitumor immune response. We propose that treatment with AU-011 may be better than other approved photosensitizers (e.g., Foscan) given that the composition of AU-011 involves a Virus Like Particle (VLP) that provides targeting specificity to tumor cells. In addition, the VLPs are known to have immunomodulatory activities that might help change the tumor micro-environment and improve the synergy with checkpoint blockade antibodies. We propose that the combination with immunotherapy will enable the treatment of distant tumors by enhancing the AU-011-induced antitumor immune response. In the present study, we have explored the feasibility of this strategy by comparing the efficacy of AU-011 treatment with several checkpoint blockade antibodies, to determine an optimal treatment regimen. We first show that AU-011 preferentially associates with cancer cells over APCs and that AU-011 treatment preferentially kills cancer cells over APCs when co-cultured. In addition, we show that AU-011 binding and AU-011 induced cytotoxicity is comparable in most, but not all, cells in a panel of murine and human colon cancer cell lines. Moreover, we report that treatment with AU-011 induces immunogenic cell death through exposure of calreticulin and release of HMGB-1 to a level that exceeds freeze thawing of the cells, suggesting that treatment with AU-011 facilitates upregulation of these DAMPs. The AU-011-induced DAMP release was shown to result in the maturation of D1DCs to a level exceeding the toll-like receptor-ligand poly I:C. Using the MC38 murine tumor model, following intravenous injection, AU-011 accumulates in the tumors, peaking at 12 h post injection as measured by increased tumor fluorescence and reduced spread between animals when compared to intraperitoneal injection. Consistent with in vitro results demonstrating high cytotoxicity, immunogenic cell death and dendritic cell maturation, treatment with AU-011 synergizes with ICI in vivo. All animals bearing a single subcutaneous tumor that were treated with a single systemic dose of AU-011 combined with CTLA-4 or with PD-L1 together with LAG-3 antibodies had a CR. In a tumor model for local and distant tumors, AU-011 again synergized with ICI with the most efficient combination being AU-011 with PD-L1 and LAG-3, resulting in a 75% CR rate. Together, our results provide support for the feasibility and efficacy of combining targeted-PDT using AU-011 with ICI for the treatment of local and distant tumors.

Our results corroborate literature reporting that treatment with AU-011 kills tumors cells while simultaneously initiating antitumor immune responses [27, 34], whereby the treatment essentially functions as an in situ tumor vaccination strategy. Currently, there is no shortage of studies reporting on the DAMP- and immunogenic cell death- inducing capacities of PDT with different photosensitizers. And although PDT as a standalone treatment approach often appears to induce immune-mediated growth inhibition on distant tumors, it is generally insufficient to achieve durable complete respones [26, 34]. The results presented here show that the antitumor effect of AU-011 is enhanced by the combination with checkpoint blockade antibodies, with high efficacy on distant tumors. The data confirms observations from studies combining other forms of PDT in combination with ICI in preclinical distant tumor models [25, 35, 36], but outperforms approaches that combine PDT with immunotherapy other than ICI against distant tumors [34]. The results also confirm recently published data [27], showing synergy of AU-011 and ICI with complete responses in mice bearing a single TC-1 tumor and showing an immune mediated long-lasting protection from tumor re-challenge. Similar to our data, they report that anti-CTLA-4 enhances survival compared to anti-PD-L1 antibodies, when each is combined with AU-011. They also show that treatment with AU-011 induces an immunogenic cell death and infiltration of CD45 + cells in TC-1 tumors. Furthermore, they elegantly show an increase, albeit not statistically significant, in tumor-epitope specific T cells. Restimulation of peripheral blood mononuclear cells (PBMCs) from AU-011 PDT treated animals with irradiated TC-1 cells ex vivo showed enhanced secretion of IFN-y with IL-2 and TNF-a with IL-2 compared to the relevant controls, indicating the existence of activated tumor specific lymphocytes. Using a different tumor model of colon cancer, MC38, we expand on their data by showing the efficacy of AU-011 and ICI on distant tumors and identify the combination with anti-PD-L1 and anti-LAG-3 as the most suitable treatment regimen for both primary and distant tumors. These results could be partially explained by the prior observation that anti-PD-L1 treatment induces the expansion of tumor-infiltrating CD4^+^ and CD8^+^ T-cell subsets that co-express activating (ICOS) and inhibitory (LAG-3, PD-1) molecules [28].

In patients, the combination of ICI and PDT has not been investigated thoroughly, although a phase I trial that investigates intraoperative PDT using porfimer sodium as the photosensitizer to amplify the response to immunotherapy in patients with non-small cell lung cancer was recently initiated (NCT04836429). AU-011 is a novel targeted therapy in clinical development for the treatment of small choroidal melanoma and indeterminate lesions in the eye and showed encouraging early results for the first line treatment of these patients with either intravitreal (NCT03052127) or suprachoroidal administration (NCT04417530). AU-011 is also under consideration for the treatment of other tumors that metastasize to the eye such as, breast, lung and colon cancer. In clinical trials, using a single ICI as a standalone treatment has shown limited efficacy on metastases of primary uveal melanoma [37, 38], especially compared to cutaneous melanoma where results are significantly more impressive. However, the results presented in this study indicate that combination therapy may be a better approach. In line with this, a phase II and III trial investigated a combination treatment consisting of anti-PD-1 (Nivolumab) together with anti-LAG-3 (Relatlimab) in patients with metastatic or unresectable cutaneous melanoma (NCT03470922) [39], showing a significant increase in progression-free survival for the combination treatment versus Nivolumab alone. As LAG-3 and its ligands display higher expression in patients with high-risk uveal melanoma [40], using anti-LAG-3 antibodies may be an effective approach for patients with metastatic uveal melanoma. Early diagnosis of uveal melanoma has improved dramatically over the years and most patients have no metastatic disease confirmed at the time that the primary tumor is identified. Unfortunately, despite local treatment with radiotherapy, patients experience dramatic vision loss in addition to disfiguration, and still have a high risk of dying from metastatic disease. Based on our observations and recent clinical data, we propose that treatment with AU-011 of a primary tumor may enhance a systemic immune response against distant tumors. Especially the use of anti-PD-L1 and anti-LAG-3 in combination with AU-011 may enhance the therapeutic outcome in patients with both primary and metastatic tumors.

## Supplementary Information

Below is the link to the electronic supplementary material.

Supplementary file1 (DOCX 1929 KB)
