# Supplementary material for: Immune checkpoint inhibition combined with targeted therapy using a novel virus-like drug conjugate induces complete responses in a murine model of local and distant tumors
Source: Cancer Immunol Immunother. 2023 Mar 30;72(7):2405–22. doi: 10.1007/s00262-023-03425-3 (PMC10264500; doi:10.1007/s00262-023-03425-3)
Supplement: Supplementary file 1 — Supplementary file1 (DOCX 1929 KB) [file 262_2023_3425_MOESM1_ESM.docx]

**Supplemental information**


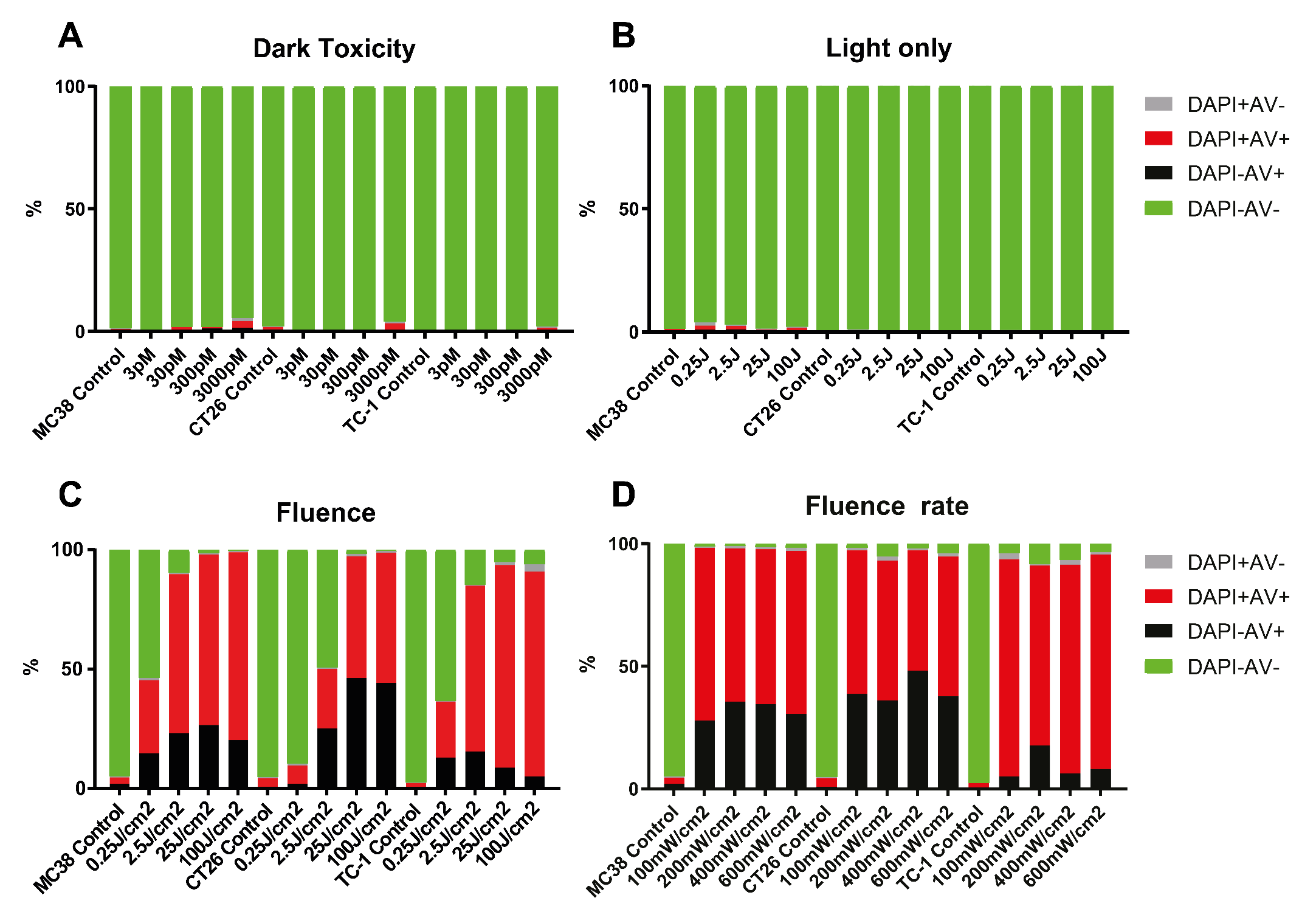


**Figure S1 Photodynamic therapy with AU-011 preferentially induces cytotoxicity in cancer cells over APCs**

(A) MC38, CT26 or TC-1 were incubated with 3-3000 pM of AU-011 in the dark for 4 h. At 18 h after treatment, the samples were stained with viability markers Annexin V-FITC and DAPI before analysis by flow cytometry. (B) MC38, CT26 or TC-1 were incubated with culture medium in the dark for 4 h. Cells were then illuminated with 690 nm light at 400 mW/cm^2^ for 25 J/cm^2^. At 18 h after treatment, the samples were stained with viability markers Annexin V-FITC and DAPI before analysis by flow cytometry. (C) MC38, CT2,6 and TC-1 were incubated with 300 pM of AU-011 in the dark for 4 h. Cells were then illuminated with NIR light at 400 mW/cm2 for 0.25-100 J/cm2. At 18 h after treatment, the samples were stained with viability markers Annexin V-FITC and DAPI before analysis by flow cytometry. (D) MC38, CT26 or TC-1 were incubated with 3-3000 pM of AU-011 in the dark for 4 h. Cells were then illuminated with 690 nm light at 600, 400, 200 or 100 mW/cm^2^ for 25 J/cm^2^. At 18 h after treatment, the samples were stained with viability markers Annexin V-FITC and DAPI before analysis by flow cytometry. (mean; n = 3).


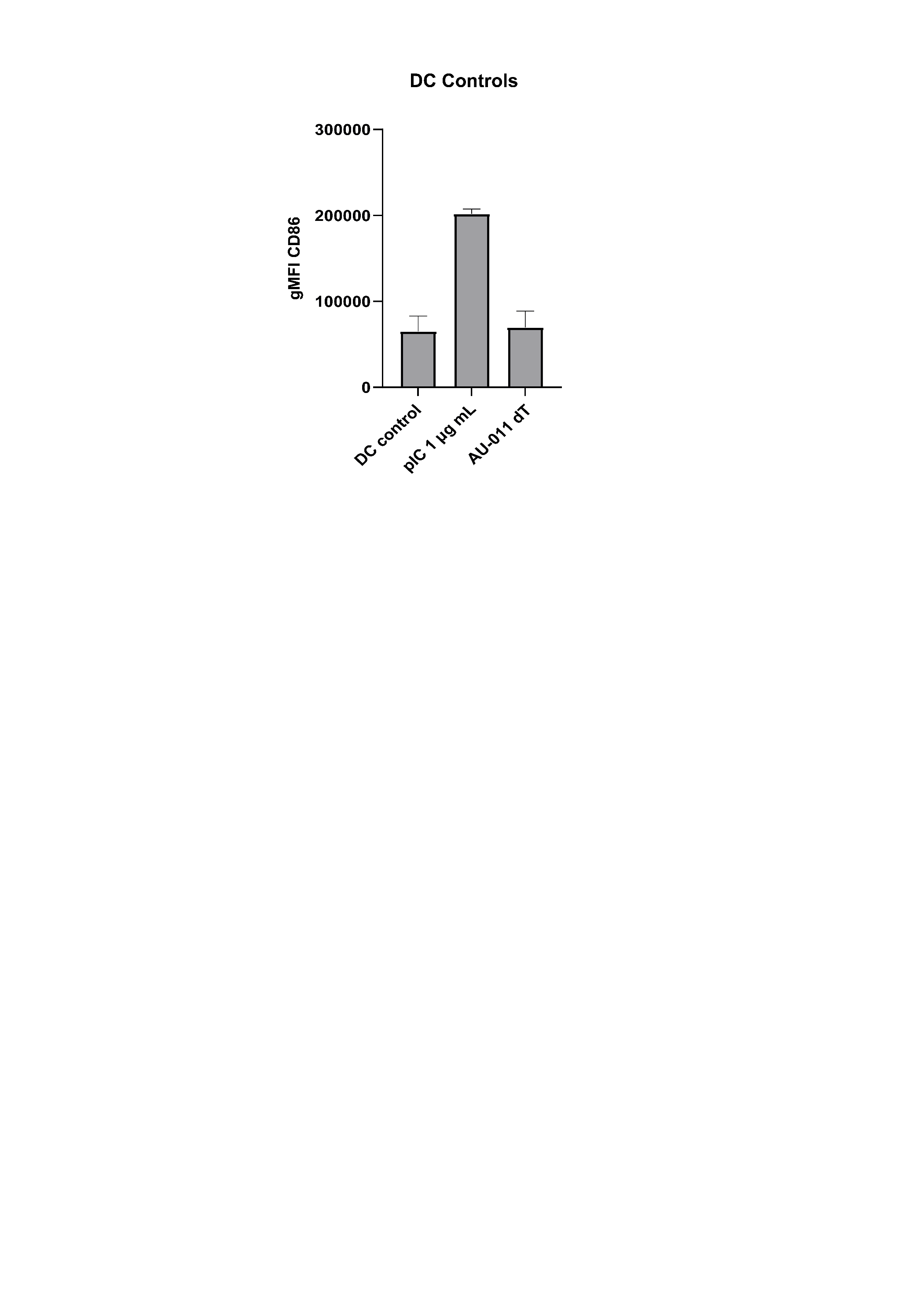


**Figure S2 D1 dendritic cell controls**

D1DCs were incubated with 300 pM AU-011 for 4 h, washed and incubated for 24 h in D1DC culture medium (AU-011 dark toxicity, dT), incubated in D1DC culture medium for 4 h, washed and subsequently incubated with 1 µg / mL of pI:C for 24 h (p:IC 1 µg / mL), or incubated in D1DC culture medium for 28 h (DC control). Cells were then washed, stained with DAPI, anti-CD11c-PE and anti-CD86-FITC before analysis by flow cytometry. The data was displayed as the geometric mean fluorescence intensity (gMFI) Gating of living D1DCs was based on DAPI^-^CD11c^+^ events. (mean ± SEM; n = 3).


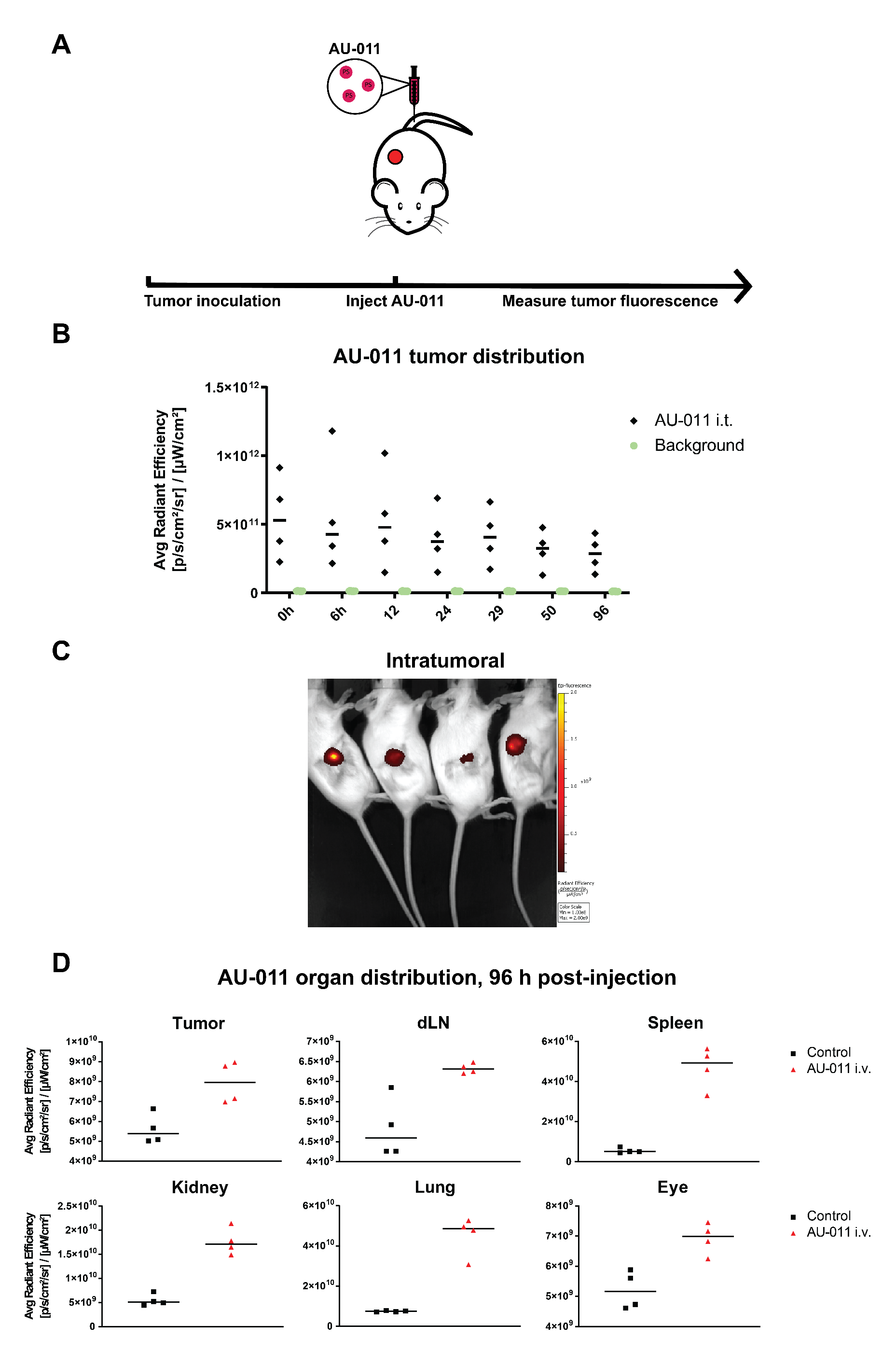


**Figure S3 Biodistribution of AU-011 after intratumoral administration in murine models**

(A) C57BL/6-albino mice were inoculated with 0.5 x 10^6^ MC38 cells in the right flank. At 7 days post inoculation, when tumors were established (125 mm^3^), the mice were injected with 30 µg of AU-011 intratumorally. (B) The fluorescence of AU-011 was measured in the tumors over time on the IVIS Spectrum fluorescence spectrometer and compared to untreated mice bearing MC38 tumors (background). (C) Representative IVIS images of animals injected intratumorally with AU-011 at 12 h post administration. (D) Animals inoculated as in (A) were injected intravenously with 100 µg AU-011 and sacrificed at 96 h post administration, after which the tumor-draining lymph nodes (dLN), spleen, kidneys, lung and eyes were harvested. The fluorescence of AU-011 in each organ was measured on the IVIS Spectrum fluorescence spectrometer immediately after harvesting. (mean; n = 4)


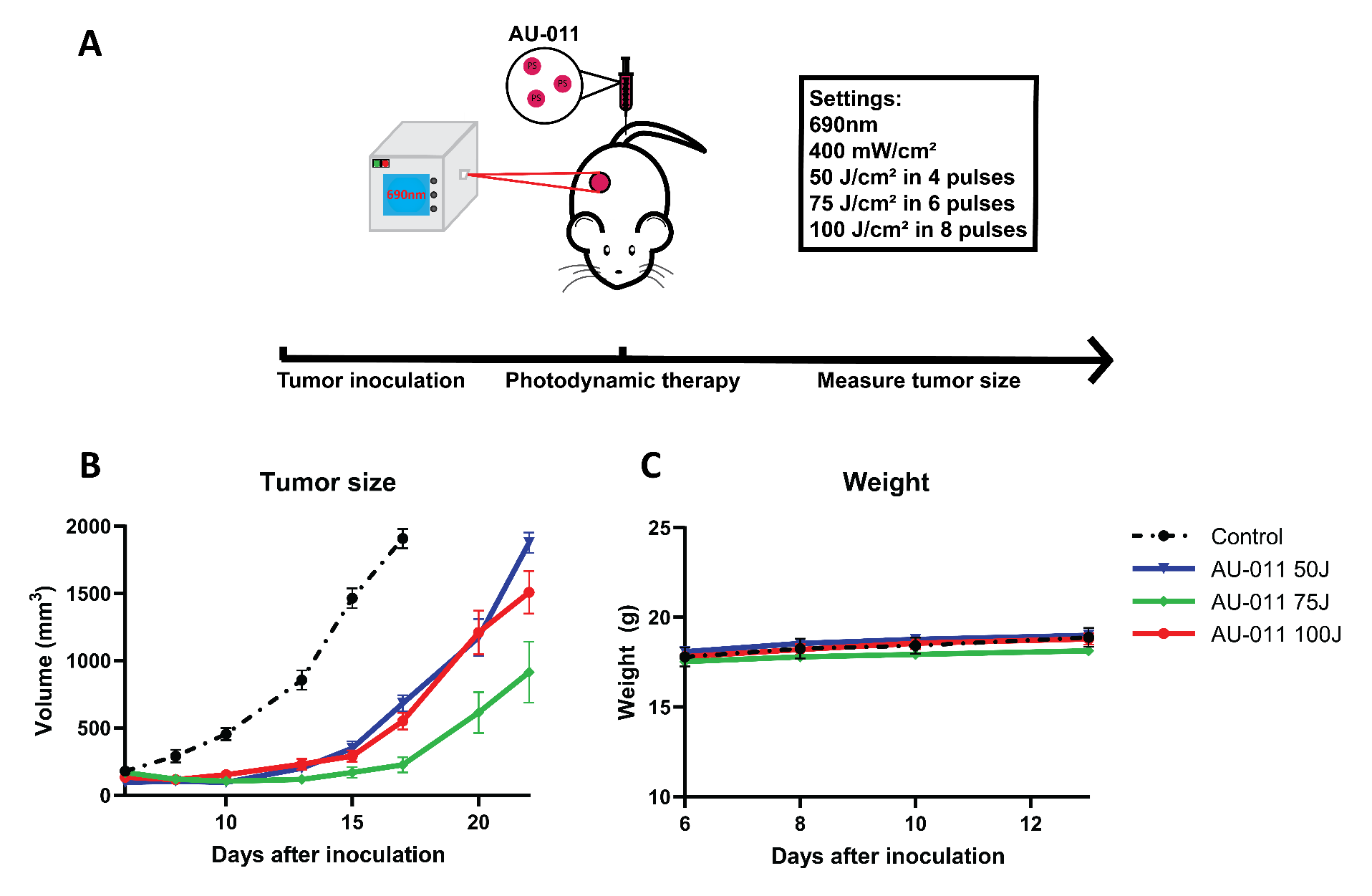


**Figure S4 Optimization of AU-011 photodynamic therapy**

(A) C57BL/6 mice were inoculated with 0.5 x 10^6^ MC38 cells in the right flank. At 7 days post inoculation, when tumors were established (125 mm^3^), the mice were injected with 100 µg of AU-011 intravenously into the tail vein. The tumors were then illuminated with 690 nm light at a DLI of 12 h with 400 mW/cm^2^ for 50 J/cm^2^ in four pulses, 75 J/cm^2^ in six pulses or 100 J/cm^2^ in 8 pulses. (B) Tumor volume of the animals after inoculation, (C) animal weight corresponding to the protocol as described. (mean ± SEM; n ≥ 7)


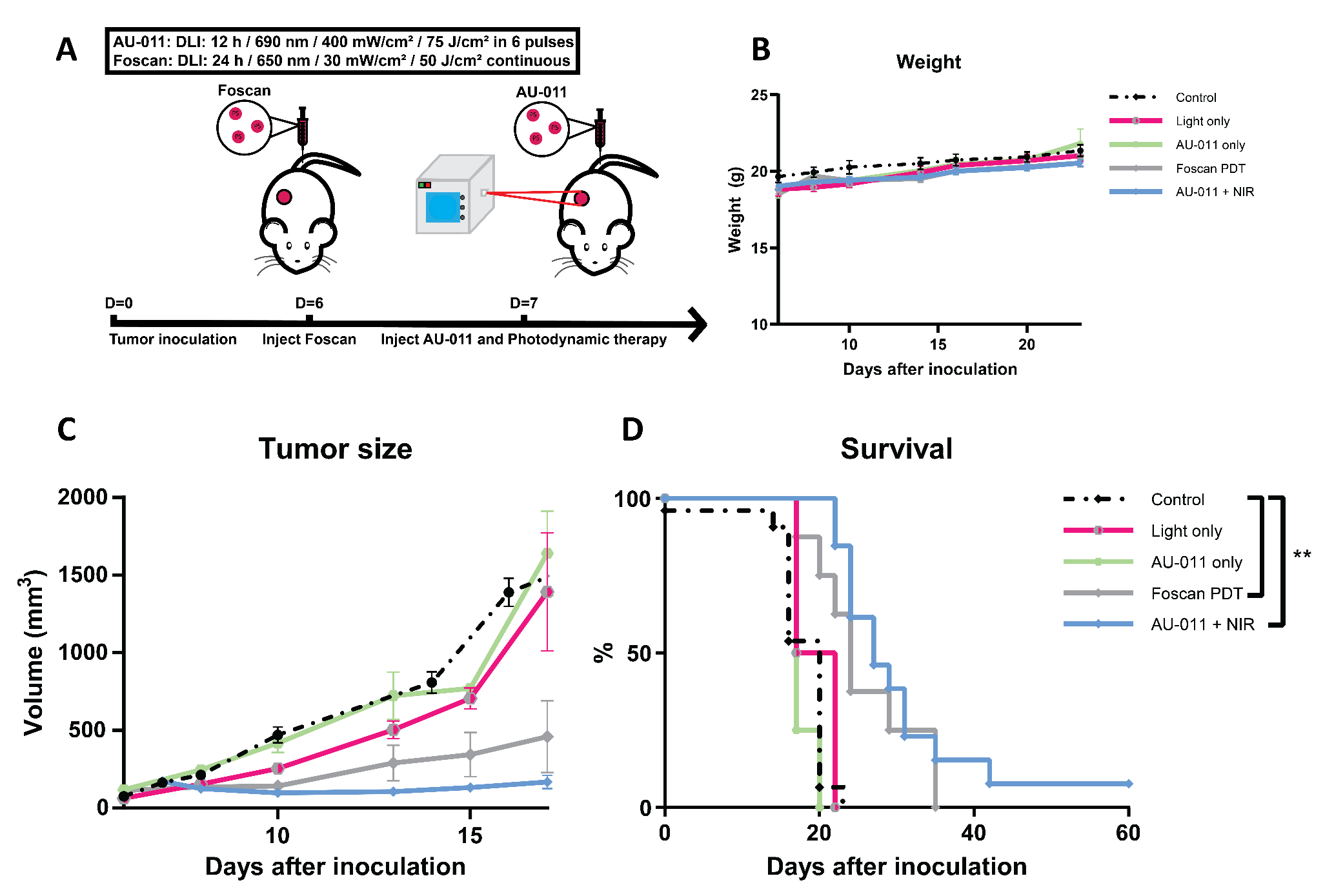


**Figure S5 Optimization of AU-011 photodynamic therapy**

(A) C57BL/6 mice were inoculated with 0.5 x 10^6^ MC38 cells in the right flank. At day 6 after inoculation, the animals were injected intravenously into the tail vein over 4-6 minutes with 0.15 mg/kg Foscan. The tumors were then illuminated with 650 nm light at a DLI of 24 h with 26.817 mW/cm^2^ for 20 J/cm^2^. At 7 days post inoculation, when tumors were established (125 mm^3^), the mice were injected with 100 µg of AU-011 intravenously into the tail vein. The tumors were then illuminated with 690 nm light at a DLI of 12 h with 400 mW/cm^2^ for 75 J/cm^2^. For light only, tumors were illuminated with 690 nm light at 400 mW/cm^2^ for 75 J/cm^2^ and for AU-011 only, the mice were injected with 100 µg of AU-011 intravenously into the tail vein. After treatments, the animals were monitored over time. (B) Animal weight, (C) tumor volume and (D) survival curves of the animals after inoculation, corresponding to the protocol as described. Statistical analysis was performed using the Mantel-Cox test (** p = 0.001; mean ± SEM; n ≥ 7)


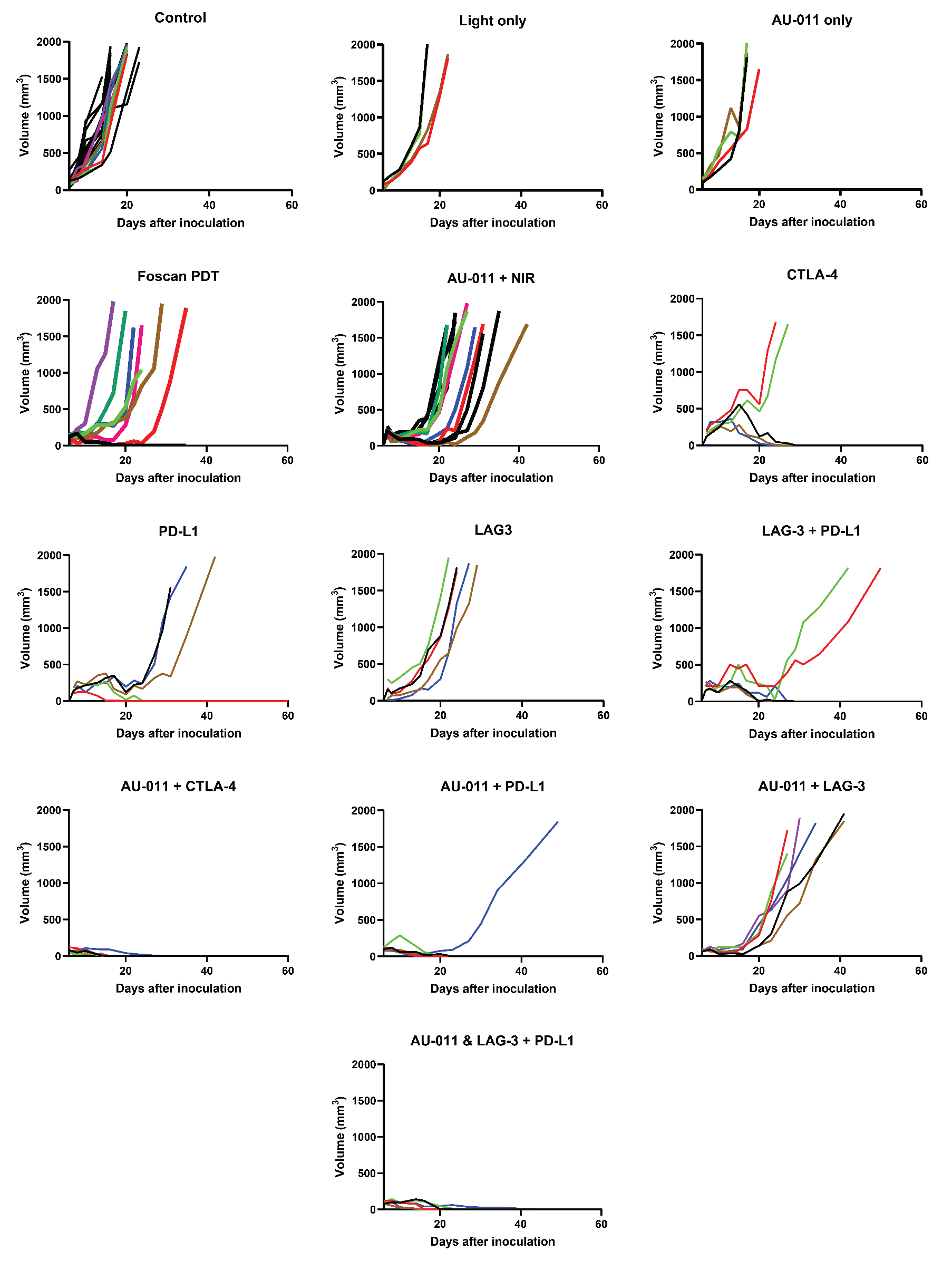


**Figure S6 Tumor growth curves of photodynamic therapy and immune checkpoint inhibition in murine tumor models**

Tumor growth curves corresponding to the protocol detailed in figure S5 and figure 5.


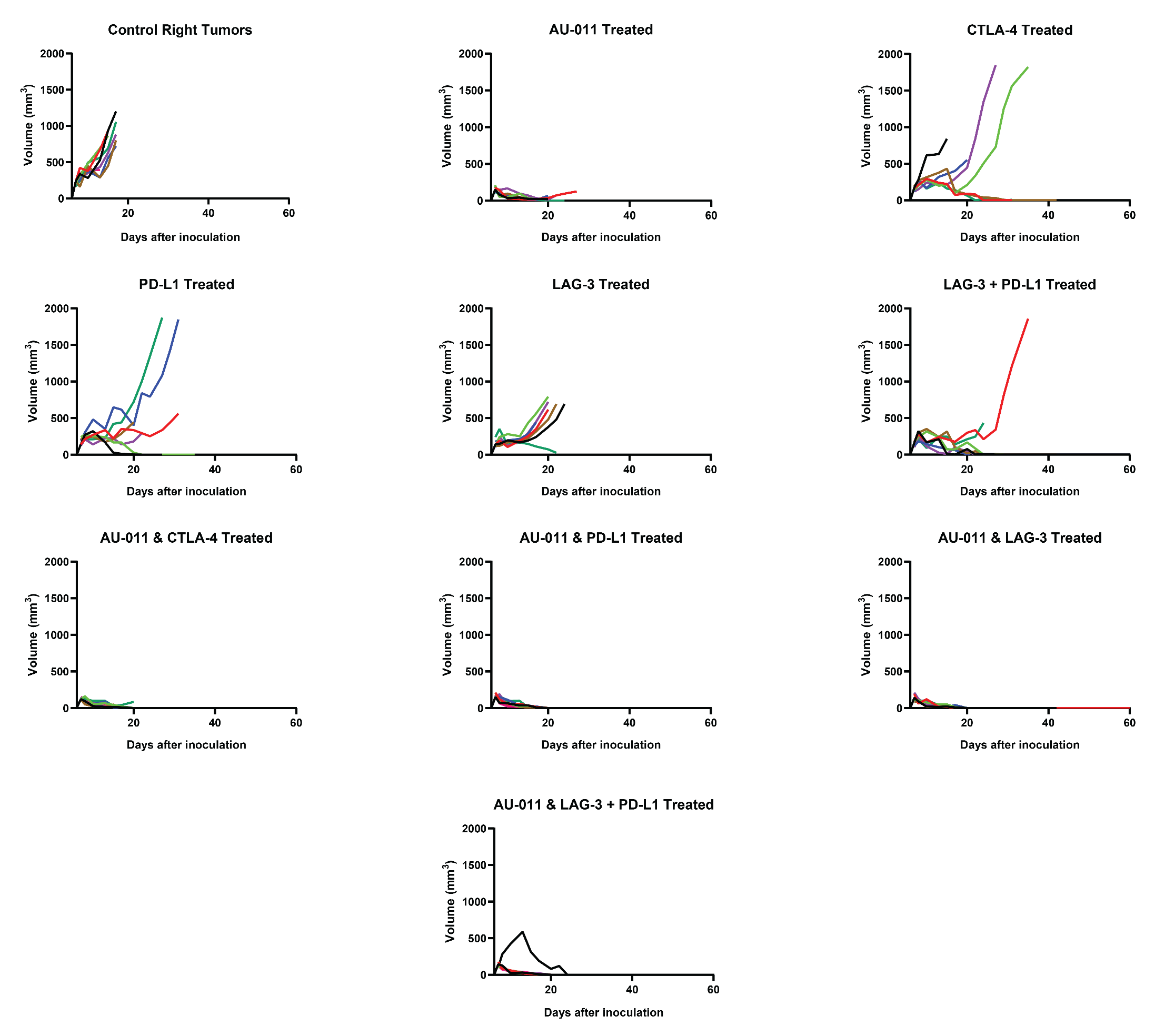


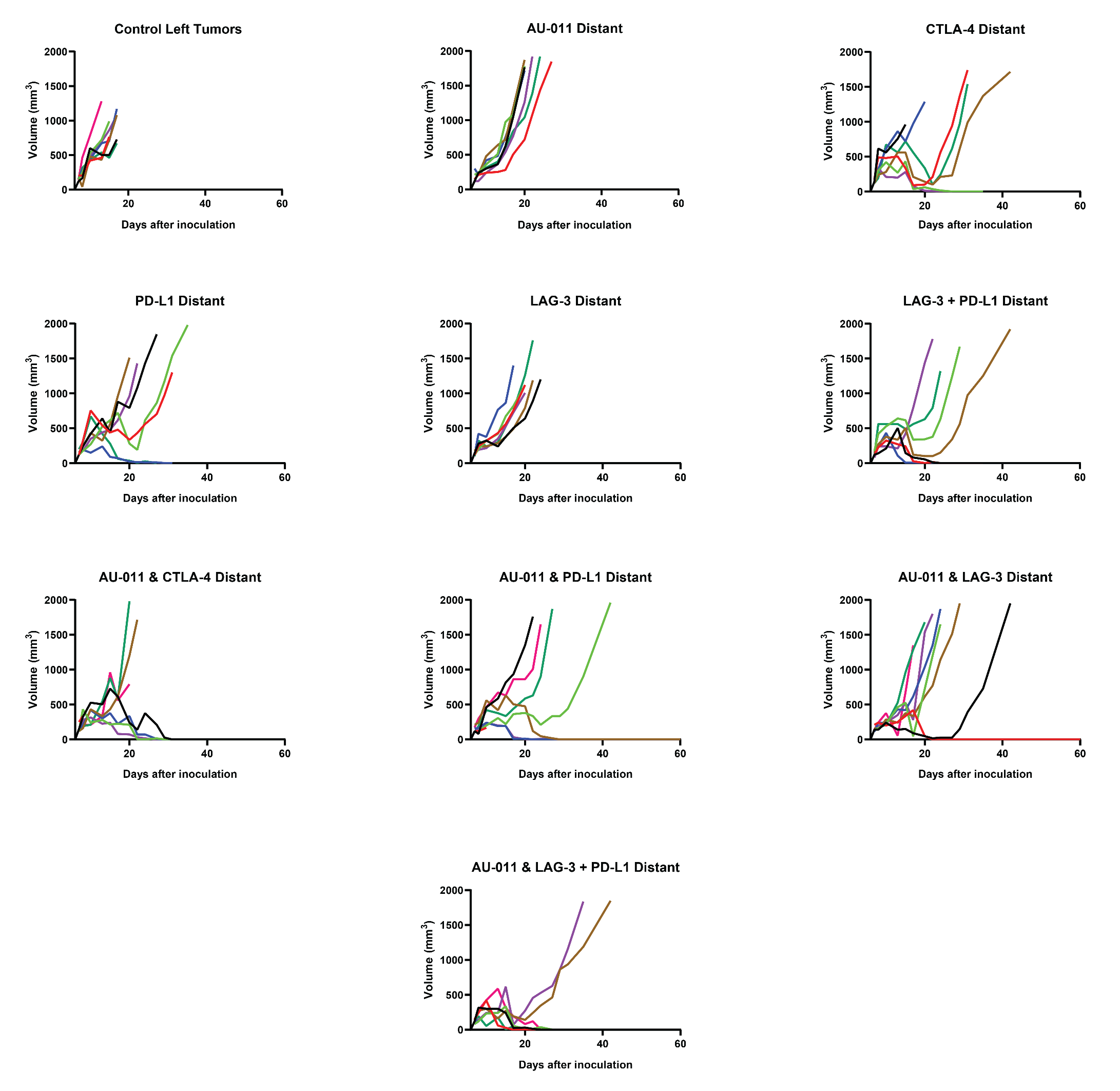


**Figure S7 Tumor growth curves of photodynamic therapy and immune checkpoint inhibition in primary and distant tumors**

Tumor growth curves corresponding to the protocol detailed in figure 6.


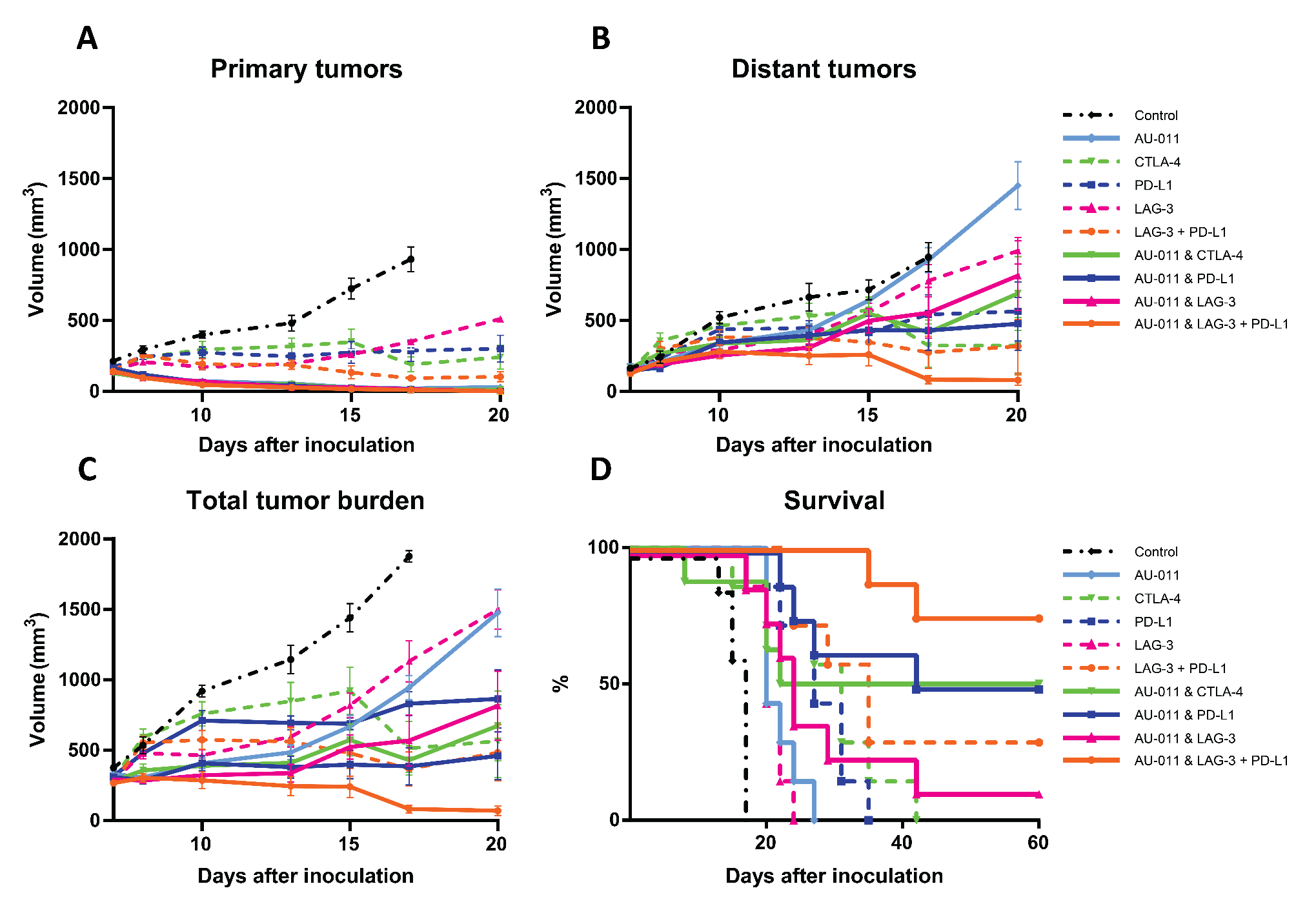


**Figure S8 Summary of photodynamic therapy and immune checkpoint inhibition in primary and distant tumors**

Summary of tumor growth curves of (A) primary (treated) tumors, (B) distant (untreated) tumors, (C) the total tumor burden (cumulative of primary and distant tumors) and (E) survival curves corresponding to the protocol detailed in figure 6.
